# Ultra-fast green hydrogen production from municipal wastewater by an integrated forward osmosis-alkaline water electrolysis system

**DOI:** 10.1038/s41467-024-46964-8

**Published:** 2024-03-23

**Authors:** Gabriela Scheibel Cassol, Chii Shang, Alicia Kyoungjin An, Noman Khalid Khanzada, Francesco Ciucci, Alessandro Manzotti, Paul Westerhoff, Yinghao Song, Li Ling

**Affiliations:** 1 Department of Civil and Environmental Engineering, The Hong Kong University of Science and Technology, Hong Kong SAR, China; 2 Hong Kong Branch of Chinese National Engineering Research Center for Control & Treatment of Heavy Metal Pollution, The Hong Kong University of Science and Technology, Hong Kong SAR, China; 3 School of Energy and Environment, City University of Hong Kong, Hong Kong SAR, China; 4 NYUAD Water Research Center, New York University Abu Dhabi, Abu Dhabi, United Arab Emirates; 5 Department of Mechanical and Aerospace Engineering, The Hong Kong University of Science and Technology, Hong Kong SAR, China; 6 Chair of Electrode Design for Electrochemical Energy Systems, University of Bayreuth, Bayreuth, Germany; 7 Nanosystems Engineering Research Center for Nanotechnology-Enabled Water Treatment, School of Sustainable Engineering and The Built Environment, Arizona State University, Tempe, AZ USA; 8 Advanced Interdisciplinary Institute of Environment and Ecology, Beijing Normal University, Zhuhai, China

**Keywords:** Environmental chemistry, Renewable energy, Energy

## Abstract

Recent advancements in membrane-assisted seawater electrolysis powered by renewable energy offer a sustainable path to green hydrogen production. However, its large-scale implementation faces challenges due to slow power-to-hydrogen (P2H) conversion rates. Here we report a modular forward osmosis-water splitting (FOWS) system that integrates a thin-film composite FO membrane for water extraction with alkaline water electrolysis (AWE), denoted as FOWS_AWE_. This system generates high-purity hydrogen directly from wastewater at a rate of 448 Nm^3^ day^−1^ m^−^^2^ of membrane area, over 14 times faster than the state-of-the-art practice, with specific energy consumption as low as 3.96 kWh Nm^−3^. The rapid hydrogen production rate results from the utilisation of 1 M potassium hydroxide as a draw solution to extract water from wastewater, and as the electrolyte of AWE to split water and produce hydrogen. The current system enables this through the use of a potassium hydroxide-tolerant and hydrophilic FO membrane. The established water-hydrogen balance model can be applied to design modular FO and AWE units to meet demands at various scales, from households to cities, and from different water sources. The FOWS_AWE_ system is a sustainable and an economical approach for producing hydrogen at a record-high rate directly from wastewater, marking a significant leap in P2H practice.

## Abstract (web summary)

Green hydrogen production faces increased water risks due to scarce supplies of water. Here, authors develop a modular forward osmosis-water splitting system that utilises wastewater effluent to generate high-purity hydrogen, providing a sustainable solution for water and energy security.

## Introduction

**Fig. 1:**
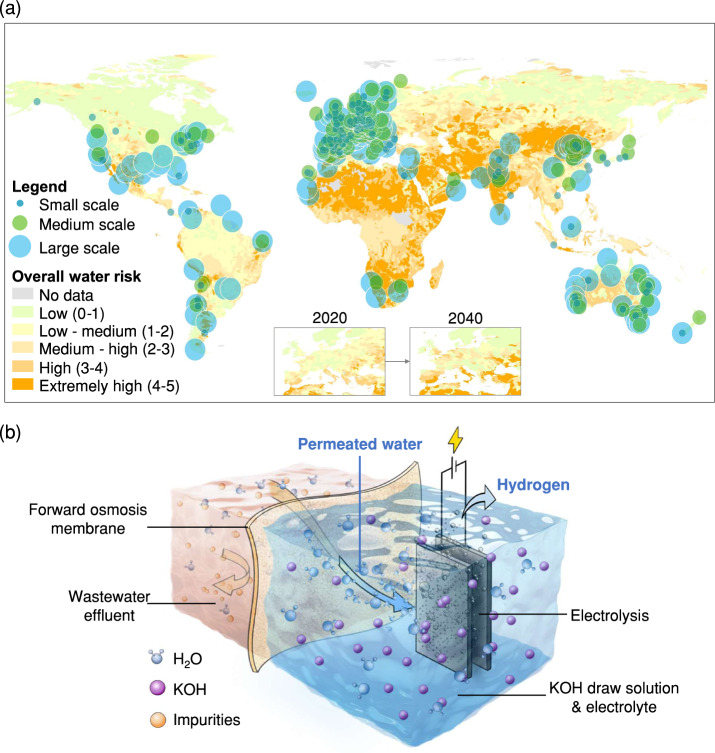
Water risk atlas with planned H_2_ projects and the FOWS_AWE_ system. **a** The distribution of hydrogen projects worldwide is categorised in small-scale (<100 MW), medium-scale (100–1000 MW), and large-scale (>1000 MW) according to water risk. The map was built using Geographic Information System (GIS) software by combining data from two global databases, i.e. water-related risk information across large geographies from the World Resources Institute (WRI) Aqueduct^55^ and a worldwide database of hydrogen projects compiled by the International Energy Agency (IEA)^56^ **b** Schematic diagram illustrating the FOWS_AWE_ system for green hydrogen production from municipal wastewater effluent.

The utilisation of renewable energy in the process of water electrolysis to produce green hydrogen offers significant promise for decarbonisation in many sectors. It is projected that by the year 2050, around 86% of the entire global annual power generation, amounting to 55,000 TWh, will be derived from renewable energy sources^1^. However, it is worth noting that up to 40% of the overall renewable electricity may be wasted due to the intermittent and variable characteristics inherent in renewable energy production^2–4^. The power-to-hydrogen (P2H) system provides a flexible approach to use renewable energy to produce green hydrogen (H_2_) via water electrolysis to minimise renewable energy wastage. Currently, over 400 P2H projects are being planned or constructed using renewable energy, mainly in European countries (comprising ~70%) (Fig. 1a), for completion by 2030. P2H projects are also expected by 2050 in North African countries, India, China, Chile, Australia, and Saudi Arabia, driven by their abundant renewable resources and diverse strategies towards a low-carbon economy^1,5,6^. However, to limit global warming to well below 2 °C and achieve climate neutrality by 2050^7^, electrolyser capacity must increase 6000–8000-fold^7,8^. Such high green H_2_ yields to meet city-scale energy require large amounts of water (i.e. 10‒15 L of water per kg of H_2_). Water is particularly valuable and crucial in areas prone to the risks of water scarcity or quality deterioration, including Northern China, the Middle East, North Africa, and India (Fig. 1a)^9^. However, it is noteworthy that around 30% of ongoing or planned P2H projects are located in regions where populations facing water scarcity are projected to increase up to 154% by 2050^10^. These issues pose a potential constraint on the wide application of the P2H strategy^11^.

Several strategies propose alternative water sources, mostly seawater, for utilisation in P2H systems. Direct seawater electrolysis results in limited electrolyser lifespan and low H_2_ production rates due to various factors including the precipitation of cations such as calcium and magnesium at the cathode, electrode corrosion, and the formation of parasitic chlorine by-products at the anode^12–15^. Seawater desalination via reverse osmosis (RO) before electrolysis is often employed for H_2_ production, but it is hampered by membrane fouling^16,17^. Incorporating forward osmosis (FO) before RO (FO–RO) mitigates membrane fouling, but it increases costs due to the secondary processes required to separate the clean water and regenerate the draw solution^17–19^.

Recent advancements in membrane-assisted seawater electrolysis offer a promising future for P2H solutions. By incorporating a hydrophobic polytetrafluoroethylene (PTFE) membrane, water can be extracted from seawater as vapour which subsequently undergoes hydrolysis^20^. Similarly, coupling FO to water electrolysis (FOWS) can extract water under an osmotic gradient, and the extracted water is sequentially hydrolysed to sustain continuous operation^21^. Such innovative systems eliminate the need for energy-intensive RO and the additional steps to separate clean water before entering the electrolyser, thus reducing capital and operational costs (Supplementary Table 2)^17,21,22^. However, both systems yield H_2_ at <30 Nm^3^ day^–1^ m^−^^2^ of membrane area, due to the low vapour pressure gradient driving the water flux across the PTFE membranes, the low conductivity of pH-neutral draw solutions in FO for electrolysis, and the high osmolarity of seawater, consequently restricting fast P2H conversion and its large-scale application^21–23^. We hypothesise that utilising potassium hydroxide (KOH) for FOWS is more efficient because KOH ensures high current densities to split the water via alkaline water electrolysis (AWE) and may serve as a suitable draw solution for FO to provide an osmotic gradient to extract water if coupled with a KOH-compatible FO membrane. Although acidic media may also enhance FOWS, using KOH is preferred as it allows the use of cost-effective materials (i.e. nickel) and minimises chlorine by-products due to higher overpotential and slower chlorine evolution reaction (CER) kinetics^24,25^. Further, the technological maturity of AWE compared to the acidified ones ensures superior safety, reliability, and scalability. We also hypothesise that wastewater effluent as an alternative water source is better than seawater, as it enhances water production due to its lower osmolarity, presents less chloride for CER, and generates less hazardous brine which is costly for disposal^26–29^. Additionally, using wastewater effluent is more practical than seawater, especially for inland communities where water resources are scarce and seawater is unavailable but where wind and solar farms can provide green electricity to drive P2H systems^30^. This background information addresses the need to better understand the selection of FO membrane materials and electrolytes, and the impacts of impurities in wastewater effluent on the H_2_ production rate of the FOWS system, and the operational versatility and stability of the system.

Here, we describe a FOWS_AWE_ system that integrates FO and AWE utilising KOH as the shared operational solute to enable continuous H_2_ production directly from wastewater effluent (Fig. 1b), achieving an impressive H_2_ production rate of 448 Nm^3^ day^–1^ m^−^^2^ of membrane area, over 14 times faster than the state-of-the-art seawater electrolysis^21,22^. Such fast H_2_ production arises from the employed semi-permeable thin-film composite (TFC)-FO membrane, which tolerates KOH concentrations up to 1 M, allowing the use of KOH as a draw solution to enhance water production from wastewater and as the optimal electrolyte to produce H_2_. The FOWS_AWE_ system also shows consistent stability, maintaining a stable cell voltage of 1.79 ± 0.01 V during the entire testing period and a high Faradaic efficiency of ~99% even with some impurities from wastewater permeating through the FO membrane. It achieved a specific energy consumption (SEC) (i.e. average energy consumption for producing Nm^3^ of H_2_) of ~4.43 kWh Nm^–3^ at 23 °C, which is comparable with commercial alkaline electrolysis using deionised water, and further reduced to 3.96 kWh Nm^–3^ at 40 °C. Moreover, the highly modular FO and AWE units, in combination with our developed water-hydrogen balance model, allow for equalising the rates of water production from FO and those of water consumption from AWE. This flexibility allows the FOWS_AWE_ system design to meet various scales with different water sources. Thus, our approach for fast and energy-efficient H_2_ production from wastewater by the highly modularised FOWS_AWE_ system demonstrates a promising strategy for the large-scale and sustainable P2H practice.

## Results and discussion

### Producing clean water via FO using KOH as a draw solution

To integrate FO with AWE, KOH and two other electrolytes including sodium bicarbonate (NaHCO_3_), and potassium pyrophosphate (K_4_P_2_O_7_) were considered as potential draw solutions for FO instead of the conventional NaCl, as NaCl induces a CER, which negatively affects H_2_ generation and damages both the FO membrane and electrodes^30–33^. Among these electrolytes at a concentration of 1 M, KOH shows current densities 18 and 12 times higher than those of NaHCO_3_ and K_4_P_2_O_7_ at 2 V, respectively (Supplementary Fig. 1). The higher current density of KOH represents a higher H_2_ production rate in AWE compared to the other two electrolytes. To not compromise the AWE performance, we tested if KOH could be used as the draw solution in FO for extracting water from simulated wastewater effluent containing sodium acetate (NaAc), ammonia/ammonium ions (NH_3_/NH_4_^+^), and chloride. Besides, although the current density of KOH increases with increasing concentration^34^, KOH at concentrations higher than 1 M causes the pH of the draw solution to exceed 14, which carries the risk of damaging commercial TFC-FO membranes. Therefore, we tested 1 M KOH to evaluate its impact on the TFC-FO membranes (details given in the *Methods* section).

**Fig. 2:**
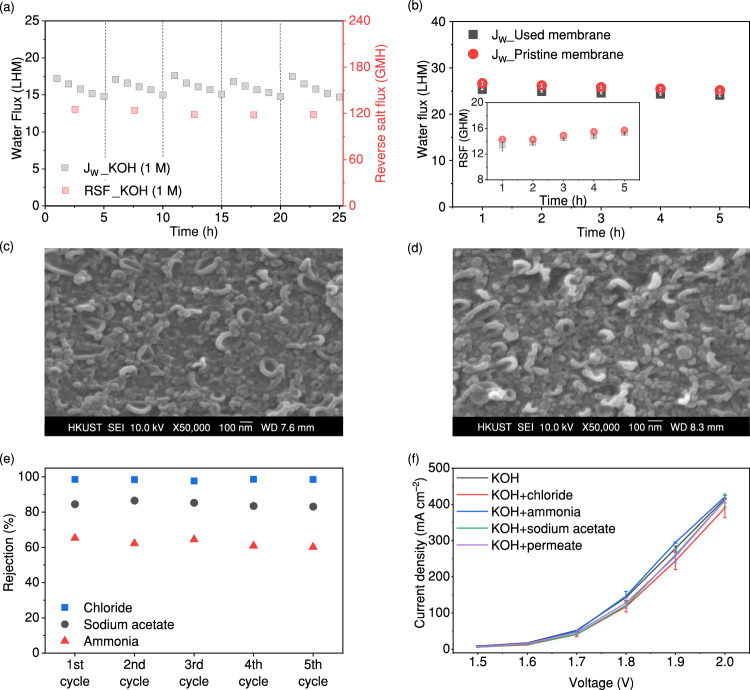
Impacts of using KOH as a draw solution of FO processes to extract clean water from wastewater effluent. **a** Water flux (*J*_w_) and reverse salt flux (RSF) of the FO process with simulated wastewater effluent containing sodium acetate, NH_3_/NH_4_^+^, and chloride as feed solution and 1 M KOH as draw solution for five cycles. The dashed lines represent five cycles of 5 h each. (Conditions: [NaAc] = 7.5 mM, [NH_3_/NH_4_^+^] = 2.2 mM as N, [Cl^–^] = 4.2 mM, and flow rate = 70 ml min^–1^); **b** comparison in water flux (*J*_w_) and RSF between the pristine membrane and the 5-cycle used membrane using deionised water as feed solution and 1 M NaCl as draw solution. The inset figure shows the reverse salt flux from the two membranes (error bars represent standard deviation from two independent replicates); **c** SEM images showing the surface morphology of the selective layer on the pristine membranes; **d** SEM images showing the surface morphology of the selective layer on the 5-cycle used membrane; **e** the rejection of sodium acetate, ammonia, and chloride by the FO membrane using 1 M KOH as draw solution (conditions: [Sodium acetate] = 7.5 mM, [Ammonia] = 2.2 mM as N, [Chloride] = 4.2 mM, and flow rate = 70 ml min^–1^); **f** J–V curves for 1 M KOH and 1 M KOH mixed with different impurities including sodium acetate, ammonia, and chloride (error bars represent standard deviation from three independent replicates). Source data are provided as a Source Data file.

Figure 2a shows the water flux (i.e. the permeated water volume per unit of time and per unit of membrane areas, L h^–1^ m^–2^ (LHM)) and reverse salt flux (RSF) (i.e. draw solute permeated to the feed solution side per unit of time and per unit of membrane areas, g h^–1^ m^–2^ (GHM)) of the FO process using 1 M KOH as the draw solution for five cycles of five hours each. The water flux decreased from 17.2 ± 0.3 to 14.9 ± 0.1 LHM after 5 h in each cycle, while the RSF remained constant at ~121 ± 3 GHM. The water flux is comparable to a system using NaCl as a draw solution (Supplementary Fig. 2), indicating that the KOH is an effective draw solution to extract water through FO processes. The primary concern associated with the use of KOH as a draw solution is its potential to damage the FO membrane, as the reverse flux of KOH may cause hydrolysis of the polymer chains that constitute the membrane selective layer (on the feed side)^35^. The integrity of the commercial TFC-FO membrane after operating in 1 M KOH solution was examined by comparing the water flux and RSF of the used membrane after the tests shown in Fig. 2a and a new pristine membrane, both using deionised water as the feed solution and 1 M NaCl as the draw solution (Fig. 2b). Figure 2b shows that the water flux of the used membrane was reduced by less than 5% compared to the pristine membrane, while the RSF of the two membranes was comparable. The membrane integrity in terms of water flux and RSF was not compromised. In addition, results from scanning electron microscopy (SEM) revealed no change in the “leaf-like” surface of the selective layer on the unused membrane and the used membrane after five cycles (Fig. 2c, d and Supplementary Fig. 3a, b) and in the peaks of the functional groups in the Fourier transform infra-red spectroscopy (FTIR) (Supplementary Fig. 4). The comparable water flux and RSF between the pristine and the used membrane suggest that the FO membrane was not damaged by KOH. The high RSF may be attributed to the smaller hydrated ionic size and higher ionic mobility of KOH^36^, or the ionisation of the membrane via deprotonation of the functional groups on its surfaces facilitating the transport of cations across the membrane^37,38^. Nonetheless, the RSF accounted for less than 1.5% of the initial KOH concentration after a cycle, suggesting the impact due to this issue was minor. Moreover, since KOH is inexpensive, any losses can be easily replenished in the system.

Since impurities in the wastewater effluent may permeate through the FO membrane and thus affect electrolysis, we examined the rejection of common impurities in the wastewater effluent by the FO membrane, including NaAc, NH_3_/NH_4_^+^, and chloride (Fig. 2e). The rejection rates of NaAc, NH_3_/NH_4_^+^, and chloride remained stable throughout the five cycles at about 85%, 63%, and 98%, respectively. The lower rejection of NH_3_/NH_4_^+^ occurred as the TFC-FO membrane surface is negatively charged, which allows anions to be intercepted more readily than cations or unionised NH_3_ which is the dominant form of NH_3_/NH_4_^+^ at high pH. The rejection of additional common impurities found in wastewater and varied pH levels was also examined, with rejection rates exceeding 90% (Supplementary Figs. 5 and 6). The potential impacts to the electrolysis process from the permeated impurities were then investigated using a commercial 5-cell alkaline stack with nickel alloy-based electrodes by comparing the current density–voltage (J–V) curves generated from: (i) 1 M KOH, (ii‒iv) 1 M KOH mixed separately with NaAc, NH_3_/NH_4_^+^, and chloride at a concentration of 10 mM for studying their accumulation effect, respectively, or (v) a 1 M KOH mixture consisting of 1.1 mM of NaAc, 0.7 mM NH_3_/NH_4_^+^ as N, and 0.08 mM chloride observed in the permeate from the rejection test, as shown in Fig. 2f. All tested solutions exhibited a consistent behaviour in their J–V curve performances, suggesting that the presence of NaAc, NH_3_/NH_4_^+^, and chloride in KOH minimally impacted the current densities. We infer that the high concentration of KOH, sustaining a high pH (>8), mitigated the negative effects of NaAc and NH_3_/NH_4_^+^ and suppressed side reactions such as chlorine evolution^31,39^. Therefore, the above results of the high and constant water flux, the intact FO membrane, and the minor impact of impurities permeated from the FO membranes on electrolysis suggested using KOH as the draw solution in the FO process to extract water from wastewater effluent is feasible.

### Integrating FO with AWE

**Fig. 3:**
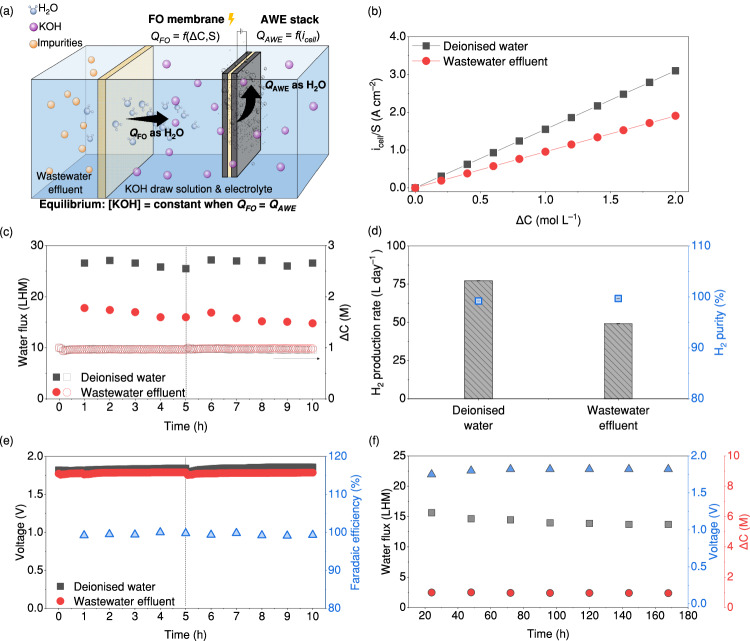
Integrating the FO processes with alkaline water electrolysis (AWE). **a** Schematics showing a dynamic equilibrium to balance the water production rate crossing the FO membrane (*Q*_FO_) and the water consumption rate by AWE (*Q*_AWE_) in the FOWS_AWE_ system; **b** the specific normalised current (*i*_cell_/*S*) as a function of the concentration gradient (Δ*C*) to reach the equilibrium in the FOWS_AWE_ system fed with deionised water and wastewater effluent. Data can be reproduced using Eq. 2; **c** the water flux and Δ*C* of the FOWS_AWE_ system during 2-cycle operation. The vertical dashed line represents the transition between the first cycle and second cycle; **d** the H_2_ production rate and H_2_ purity for using deionised water and wastewater effluent as the feed of the FOWS_AWE_ system (error bars represent standard deviation from three independent replicates); **e** the resulting voltage during the operation of the FOWS_AWE_ system. The vertical dashed line represents the transition between the first cycle and the second cycle (conditions: [KOH] = 1 M, *S* = 1 cm^2^, *i*_cell_ = 1.55 A for deionised water and 0.95 A for wastewater effluent (HK-WWE1)); **f** the water flux, voltage, and Δ*C* during the 168-h continuous operation of the integrated FOWS_AWE_ system (conditions: [KOH] = 1 M, *S* = 1 cm^2^, and *i*_cell_ = 0.90 A using the HK-WWE-2). Source data are provided as a Source Data file.

A water-hydrogen balance model was established to quantify and balance the FO and AWE units in the FOWS_AWE_ system, as shown in Fig. 3a, with the objective of continuous operation to effectively produce H_2_ from wastewater effluent. In the integrated system, clean water is first extracted from the wastewater effluent by the FO process using KOH as the draw solution and then split into H_2_ and oxygen (O_2_) by AWE using the 5-cell alkaline stack containing nickel alloy powder combined with nickel foam electrodes separated by polymer diaphragms (detailed setup is shown in the ‘Methods’ section). A sustainable operation of this system thus requires a dynamic equilibrium that enables the water production rate crossing the FO membrane (*Q*_FO_) to match the water rate consumed by AWE (*Q*_AWE_) as shown in Eq. 1, while maintaining a consistent concentration of KOH.

1 $${{Q}_{{{{{{\rm{FO}}}}}}}\,=\,S\Delta C\frac{{K}_{{{{{\rm{W}}}}}}I}{d}{{RT}}\,=\,Q}_{{{{{{\rm{AWE}}}}}}}\,=\,{i}_{{{{{{\rm{cell}}}}}}}\frac{{{{{{\rm{FE}}}}}} \, {{{\cdot} \, N}_{{{{{{\rm{cell}}}}}}}V}_{{{{{\rm{m}}}}}}}{2F}$$

*Q*_FO_ is a function of controllable parameters in the FO process including the membrane area (*S*, cm^2^) and the concentration gradient between draw solution and feed solution (Δ*C*, M)^40^. The membrane permeability coefficient (*K*_w_, L atm^–1^ s^–1^ cm^–1^), when multiplied by the van’t Hoff factor of the feed solution (*I*), represents how easily water can be extracted through the membrane, remaining constant throughout the test. Other parameters, including the membrane thickness (*d* = 0.1 mm), the ideal gas constant (*R* = 0.0821 L atm K^–1^ mol^–1^), and temperature (*T* = 296 K), also remained constant during the test. *Q*_AWE_ is only controlled by the applied current of AWE (*i*_cell_, A)^41^, as other parameters, including the Faradaic efficiency (FE), the number of cells constituting the electrolyser stack (*N*_cell_ = 5 for our setup), *V*_m_ (as the molar volume of H_2_O at 0.018 L mol^–1^ at 296 K of *T* and 1 atm), and Faraday’s constant (*F* = 96,485 C mol^–1^) are constant throughout the test. Therefore, through balancing *Q*_FO_ and *Q*_AWE_, a linear correlation was established to describe the specific normalised current of the FOWS_AWE_ system (*J*_i_, A cm^−2^), which is defined as the required current of this integrated system for handling the permeated water per area of FO membranes (*i*_cell_  *S*^–1^), changed with Δ*C* as shown in Eq. 2.

2 $${J}_{i}\,=\,\frac{{i}_{{{{{{\rm{cell}}}}}}}}{S}\,=\,\left(\frac{{K}_{{{{{\rm{w}}}}}}{\it{I}}}{d}{RT}\frac{2F}{{{{{{{{\rm{F}}}}}} \, {{{{{\rm{E}}}}}}\,N}}_{{{{{{\rm{cell}}}}}}}{V}_{{{{{\rm{m}}}}}}}\right)\Delta C\,=\,\varPhi \cdot \Delta C$$

where *Φ* is the H_2_ production potential (L A mol^–1^ cm^–2^), determined by the configurations of the FO membranes and AWE, types of feed and draw solutions, and operational conditions. H_2_ production potential (*Φ*) was calculated by substituting the values of *K*_w_ × *I*, *d*, *R*, *T*, FE, *N*_cell_, and *V*_m_ into Eq. 2, and *K*_w_ × *I* was obtained experimentally by measuring *Q*_FO_ under given conditions of *S* and Δ*C* in an independent FO module using Eq. 1, whereas FE was calculated by measuring the H_2_ production rate and purity at a given *i*_cell_ of the AWE module (detailed in the ‘Methods’ section). In this study, *Φ* values for feeding deionised water and the wastewater effluent collected from a wastewater treatment plant in Hong Kong (HK-WWE1) to the FOWS_AWE_ system were calculated as 1.55 and 0.95 A mol^–1^ cm^–2^, respectively. The lower *Φ* for wastewater effluent was attributed to impurities in the wastewater effluent reducing *K*_w_ × *I*. The obtained *Φ* values were then used to generate the relationship between *J*_i_ and Δ*C* when reaching a dynamic equilibrium for the FO and AWE units in the FOWS_AWE_ system, as shown in Fig. 3b. Therefore, to maintain Δ*C* at 1 M, *J*_*i*_ was calculated as 1.55 and 0.95 A cm^–2^ for deionised water and HK-WWE1, respectively^21^.

The two setups of FO and AWE were then experimentally integrated using a 1 cm^2^ FO membrane and applying an *i*_cell_ of AWE of 1.55 and 0.95 A for deionised water and HK-WWE1 according to their *J*_*i*_ values, respectively (integrated process shown in Supplementary Fig. 17). As shown in Fig. 3c, d, the continuous operation of this integrated system over two cycles of 5 h each, demonstrated a stable water flux crossing the FO membrane, constant Δ*C*, and a stable H_2_ production rate when fed with either deionised water or secondary wastewater treated effluent. In addition, the molar ratio of the H_2_ generation rate to *Q*_FO_ was close to one after the two test cycles (Supplementary Fig. 7), indicating that nearly all the water produced from FO was consumed by AWE. These observations indicate a successful integration of FO and AWE processes and validate our established dynamic equilibrium for the FOWS_AWE_ system. Using wastewater effluent as the feed resulted in a 38% decrease in both *Q*_FO_ and H_2_ production rate compared to feeding deionised water, which aligns with the decrease observed in the value of *K*_w_ × *I* from wastewater effluent. This indicates that these impurities mainly affect the capacity of the water produced from FO, and the ability of the FOWS_AWE_ system to produce H_2_ relies on this water production capacity. Nevertheless, using wastewater effluent as the feed did not compromise the H_2_ purity, as evidenced by the comparable H_2_ percentage observed in samples produced using both deionised water and wastewater effluent, as shown in Fig. 3d. Additionally, the stability of H_2_ production was not affected either, as the FOWS_AWE_ system operated at a near-constant voltage of 1.79 ± 0.01 V and maintained a Faradaic efficiency of over 99% throughout two cycles of 5 h each (Fig. 3e), indicating that there were no side reactions caused by impurities crossing the FO membranes or accumulation of undesired impurities.

A continuous 168-h operation of the FOWS_AWE_ system was conducted to assess its long-term stability under consistent conditions using a wastewater effluent sample collected from a second wastewater treatment plant in Hong Kong (HK-WWE2), and the result is shown in Fig. 3f. The water flux decreased by ~12% from 15.6 to 13.7 LHM, whereas the voltage increased by 4% during the initial 24 h, starting from 1.75 V and maintaining at 1.82 V. In addition, the system sustained a Δ*C* consistently at around 1 M during the entire 168-h testing period. The 12% reduced water flux after the long-term operation was attributed to fouling from organic matter in the wastewater^42^, because the ratio of C to O elements on the membrane surface was reduced by ~13%, and no degradation of integrity for both the selective and support layers of the FO membrane was observed in SEM coupled with Energy Dispersive X-ray (Supplementary Table 3 and Fig. 8, respectively). FO membrane fouling was shown to be largely reversible, unlike the often irreversible fouling challenges faced in RO^43^, which means that the reduced water flux in our system can be easily recovered by backwashing or chemical washing. Notably, the H_2_ gas purity remained stably high at over 99% throughout the entire long-term operation (Supplementary Fig. 9). These results demonstrated the operational stability of the FOWS_AWE_ system throughout the 168-h testing period. In addition, as the FOWS_AWE_ system relies on the water flux to generate H_2_, we tested more wastewater samples from the southern and northeastern regions of China, with their composition and concentrations detailed in Supplementary Table 4. The results show that our system exhibited a constant water flux, voltage, and Δ*C* for various impurities and pH values from different wastewater samples (Supplementary Figs. 10–12). Despite the varied *K*_w_ × *I* caused by different impurities, the resulting H_2_ production rates were consistent and remained within a range of 5% (Supplementary Fig. 11d). These results validate the stability and adaptability of our system for producing H_2_ under varying wastewater conditions.

### H_2_ production and energy efficiency of the FOWS_AWE_ system

**Fig. 4:**
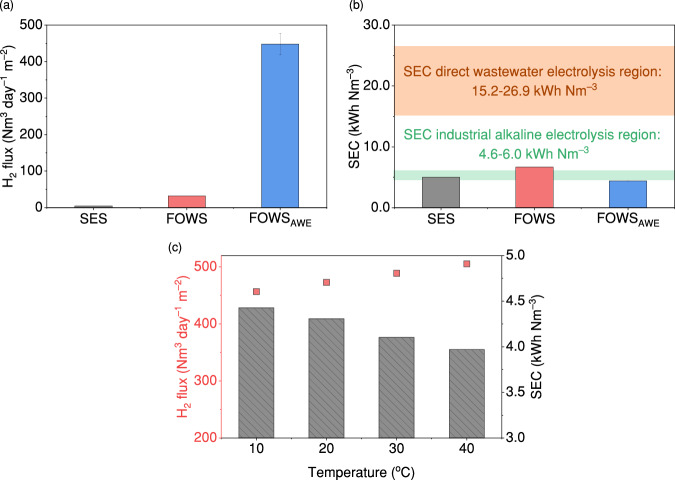
H_2_ production and energy efficiency of the FOWS_AWE_ system. **a** The comparison in H_2_ flux among three membrane-based P2H systems (error bar represents the standard deviation from three independent replicates); **b** the comparison in specific energy consumption (SEC) among three membrane-based P2H systems. The orange region represents the SEC range typical for direct wastewater electrolysis. The green region represents the SEC range for industrial alkaline water electrolysis; **c** H_2_ flux and SEC of the FOWS_AWE_ system changed with KOH temperatures. Source data are provided as a Source Data file.

The H_2_ production and energy efficiency of the FOWS_AWE_ system showed superior performance compared with two other membrane-based P2H systems for seawater purification integrated with electrolytic water splitting: (i) the seawater electrolysis system (SES) using hydrophobic PTFE membranes with 30% KOH as a self-dampening electrolyte (SDE), and (ii) the FOWS employing cellulose triacetate (CTA) membranes with 0.8 M sodium phosphate as the electrolyte^21,22^. The H_2_ produced in the three systems was compared by normalising the H_2_ production rate over the area of water-producing membranes, as shown in Fig. 4a. The FOWS_AWE_ system produced the highest H_2_ rate at 448 Nm^3^ day^–1^ m^−^^2^ of TFC-FO membrane, which is 102 and 14 times higher than those of SES and FOWS, respectively. This significant improvement in H_2_ production compared with SES is primarily due to the higher *Φ* provided by FO membranes in producing more water. Unlike SES using hydrophobic PTFE membranes, the TFC-FO membrane is hydrophilic and thus offers higher *K*_w_ to allow water to permeate. This is verified by the 23‒32 times higher *J*_*i*_ of the TFC-FO membrane to reach equilibrium than the PTFE membrane at the same values of *S*, Δ*C*, and using identical AWE setups (Supplementary Fig. 13). Besides, PTFE membranes face wetting issues during operation, which reduces the ability to reject impurities as water penetrates the pores^44,45^. The enhanced H_2_ production compared to FOWS is mainly attributed to the 5× higher Δ*C* between wastewater effluent and 1 M KOH compared to the 0.2 M Δ*C* between seawater and 0.8 M sodium phosphate, enhancing the driving force for producing water. The H_2_ flux comparison along with the water-hydrogen model suggests the water production rate dominates in determining the H_2_ flux in a membrane-electrolysis system, highlighting the importance of optimising both the membrane type and the feed water source. Moreover, high water production rates offer additional benefits of requiring fewer membranes and associated equipment, thereby reducing space and capital costs which are particularly important considerations for scaling up the system.

The FOWS_AWE_ system exhibits low energy consumption, as shown in Fig. 4b, where the SEC of the three systems is compared in addition to direct wastewater electrolysis. The FOWS_AWE_ system requires the lowest SEC at 4.43 kWh Nm^–3^ at an *i*_cell_ of 0.95 A using wastewater effluent as feed. This SEC is comparable to commercial alkaline electrolysis systems that use deionised water and is 10%, 34%, and 84% lower than SES, FOWS, and direct electrolysis of wastewater, respectively^46^. The lower SEC highlights the importance of preventing undesired impurities from electrolysis and using a pH-tolerant membrane to enable the use of KOH as an electrolyte as it has higher ionic conductivity for more efficient electron transfer. Such excellent energy efficiency is also applicable to other commercial AWE configurations, if the applied current matches the water-hydrogen equilibrium, making the FOWS_AWE_ system easily adaptable and scalable to meet varying market demands. Therefore, the hydrophilic property of TFC-FO membranes, the higher Δ*C* resulting from wastewater effluent usage instead of seawater, and the use of an optimal electrolyte (i.e. KOH) all contribute to a high H_2_ flux and energy-efficient P2H conversion.

In addition, electrolysis is an exothermic process that generates heat, so capturing and utilising waste heat during AWE operations is critical in achieving the energy-efficient operation of the system. By simulating the capture and utilisation of the waste heat during AWE operations to heat up the KOH draw solute temperature to 40 °C, the FOWS_AWE_ system shows an increase of H_2_ flux by 11% and a decrease of SEC from 4.43 to 3.96 kWh Nm^–3^ (Fig. 4c and detailed calculations provided in Supplementary Note 1). This brings us closer to the target of 3.75 kWh Nm^–3^ proposed by the International Renewable Energy Agency^47^. The enhanced H_2_ flux is mainly due to the increased water production at higher temperatures, indicated by Eq.1^48^, while the reduced SEC is attributed to the increased KOH ionic conductivity reducing the overall resistance of the cell and enabling more efficient electron transfer (Supplementary Fig. 14). These results also suggest that the FOWS_AWE_ system can perform better in hot regions where the wastewater has temperatures of up to 35 °C and can potentially heat the draw solutions during membrane operational processes^28^, or in areas with abundant solar energy that can be used for heating.

### Implications for H_2_ production using wastewater effluent

This study demonstrated the use of municipal wastewater effluent by a FOWS_AWE_ system for fast and energy-efficient green H_2_ generation to address the large-scale H_2_ production need associated with potential water constraints. The FOWS_AWE_ system exhibits stable and continuous H_2_ production at the highest yields of H_2_ to date (448 Nm^3^ day^–1^ m^–2^) using alternative water resources achieving a high H_2_ purity of >99% and low SEC of 4.43 kWh m^–3^ during the entire testing period of over 10 h. In addition, the FOWS_AWE_ system demonstrated long-term stability by being operated continuously for 168 h using real wastewater effluent and proved its capability and adaptability in producing H_2_ across diverse wastewater conditions found in different regions.

Using wastewater effluent is crucial for sustainable H_2_ production in regions where seawater is unavailable or freshwater resources are scarce, especially as H_2_ projects are rapidly expanding amid the foreseeable intensified worldwide water stress. The modular FO and AWE system along with the established water-hydrogen balance model allow the FOWS_AWE_ system to operate with different influent water quality at different scales, ensuring an easy yet versatile approach for sustainable P2H conversion from the household to the city scale. The integration of TFC-FO with electrolysis can reduce the capital costs of water treatment by up to 46% compared to conventional RO and FO–RO processes^49^, providing a strong economic incentive for FOWS_AWE_ adoption. Addressing pressing global challenges such as water scarcity, climate change, and energy insecurity requires urgent action. Existing technologies, such as the FO and AWE, can substantially impact on solving these challenges if applied and integrated more effectively. The FOWS_AWE_ system exemplifies leveraging synergistic technologies to rapidly develop economical, efficient, and sustainable solutions for interconnected environmental issues.

In addition to the H_2_ production market for industrial use and energy storage, the FOWS_AWE_ system provides advantages to wastewater treatment plants and many industries, as it serves as a tool for water reuse. The system has the potential to reduce the treatment load and wastewater discharge while simultaneously generating onsite energy, and mitigating environmental impacts^50^. It is estimated that an onsite FOWS_AWE_ station with a capacity of 5‒6 MW can produce O_2_ at ~20,000 kg day^–1^, which can supply the aerobic processes of a wastewater treatment plant in treating 50,000 m^3^ day^–1^ of wastewater (calculation shown in Supplementary Note 2)^51,52^ or support high-density in-land and offshore aquaculture^53,54^. Moreover, fuel cells that use H_2_ to generate electricity also produce high-purity water as a byproduct, which can be supplied to industries that require it, e.g. semiconductor and pharmaceutical industries.

## Methods

### Chemicals

Potassium hydroxide (KOH, Scharlau, 85.0–100.5%), sodium bicarbonate (NaHCO_3_, Sigma-Aldrich, 99.5–100.5%), potassium pyrophosphate (K_4_P_2_O_7_, Macklin, 99%), sodium chloride (NaCl, Sigma-Aldrich, ≥99%)), ammonium chloride (NH_4_Cl, Sigma-Aldrich, ≥99.5%), and sodium acetate (CH_3_COONa, Sigma Aldrich, ≥99%). The stock solutions were prepared by dissolving these chemicals in deionised water (18.2 MΩ cm) produced by a water purification system (Millipore, USA). The simulated wastewater effluent was prepared by adding 7.5 mM of sodium acetate, 2.7 mM of NH_3_/NH_4_^+^, and 4.2 mM of chloride into deionised water. Two wastewater effluent samples were collected from two different treatment plants in Hong Kong SAR labelled as HK-WWE1 and HK-WWE2. Additionally, one wastewater effluent sample was obtained from Guangdong Province, China, designated as GD-WWE, and another from Northeast China, referred to as NC-WWE. All the samples were filtered with high-pressure filtration equipment using cellulose membrane filters (pore size: 0.45 µm) and stored at 4 °C. The quality of the wastewater effluent samples used in this study are presented in the Supplementary Table 4.

### FO to extract clean water

The experiments to produce clean water from FO processes were conducted in an independent FO module as shown in Supplementary Fig. 15. The FO module consists of an FO cell with two chambers with an area of 12 cm^2^ and a depth of 0.3 cm separated by a single flat FO membrane sheet (051303, Porifera). The feed solution chamber was connected to a feed solution tank with a volume of 500 mL while another was connected to a draw solution tank with a volume of 200 mL. The feed solution was recirculated from the feed tank to the FO feed solution chamber by a peristaltic pump at a flow rate of 70 mL min^–1^. On the other side, the draw solution was recirculated from the draw solution tank to the FO draw solution chamber at the same flow rate. The water flux (*J*_W_, LHM) was gravimetrically measured by the loss of water weight in the feed tank per unit of time using an electronic balance (FX-3000GD, A&D Instruments) divided by the membrane area (Eq. 3).

3 $${J}_{{{{{\rm{W}}}}}}\left({{{{{\rm{LHM}}}}}}\right)\,=\,\frac{\Delta M}{\rho \, {\cdot} \, S \, {\cdot} \, \Delta t}$$

where Δ*M* is the water weight change during the test (g), *ρ* is the water density of 1 kg L^–1^, *S* is the membrane area (m^2^), and Δ*t* is the time interval of recording water weight (h). The RSF was determined by measuring the potassium ion concentration via an Inductively Coupled Plasma-Optical Emission Spectrometer (ICP-OES, 725-ES, Varian). The RSF (*J*_S_, GHM) was obtained by the changes in KOH concentration to the feed solution, as shown in Eq. 4.

4 $${{{{{\rm{RSF}}}}}}\left({{{{{\rm{GHM}}}}}}\right)\,=\,\frac{{V}_{t}\cdot{C}_{t}\,-\,{V}_{0}\cdot{C}_{0}}{{S}{\cdot}\Delta t}$$

where *V*_0_ and *V*_t_ represent the volumes of the feed solution before and after testing, while *C*_0_ and *C*_t_ represent the concentrations of KOH in the feed solution before and after the tests, respectively. The conductivity and the pH of the feed solution were monitored using a multiparameter benchtop metre (Multi 9630 IDS, WTW inoLab). The rejection of impurities by the FO membrane was calculated using Eq. 5:

5 $${{{{{\rm{Rejection}}}}}} \, \left(\%\right)\,=\,\left(1\,-\,\frac{{C}_{{{{{\rm{P}}}}}}}{{C}_{{{{{\rm{f}}}}}}}\right)\,\times\,100$$

where *C*_p_ and *C*_f_ represent the concentrations of the permeated impurities including sodium acetate, NH_3_/NH_4_^+^, or chloride through FO membrane and their initial feed concentrations, respectively. The concentrations of sodium acetate and NH_3_/NH_4_^+^ were measured by a total organic carbon analyser coupled with a total nitrogen measurement (TOC-L analyser with TNM-L, Shimadzu). The concentrations of NH_3_/NH_4_^+^ in the wastewater effluent samples were measured by spectrophotometric method. The concentrations of chloride were quantified using ion chromatography (IC, 940 Professional IC Vario, Metrohm). The membrane before and after use was characterised by SEM (JSM-6700F, JEOL and MAIA3, Tescan) and FTIR (Vertex 70, Bruker).

### Water electrolysis system to produce hydrogen

The experiments to produce H_2_ were conducted in an independent AWE module, as shown in Supplementary Fig. 16. The AWE module consists of a 5-cell alkaline stack (LBE-5SC, Light Bridge Inc.) that utilises bipolar electrodes separated by polymer diaphragms, an electrolyte supply tank, and a gas separator for collecting H_2_ and O_2_. The electrodes, that are composed of nickel alloy powder combined with nickel foam, have a thickness of 0.8 mm, a diameter of 44 mm, an area of 15.2 cm^2^, and a mass loading of 74.3 mg cm^–2^. Meanwhile, the polymer separators have a thickness of 100–130 µm, a diameter of 51 mm, and an area of 15.2 cm^2^. The lower potential cut-off was determined to be 1.5 V per cell. The alkaline stack was supplied with different electrolytes using a peristaltic pump. The J–V curves of different electrolytes were collected using a DC power supply (DP3030, MESTEK). The impact of impurities on water electrolysis was determined by adding the target compounds in the electrolyte to obtain J–V curves. The impact of temperature on water electrolysis was obtained by regulating the electrolyte temperature using a water bath (HH-1, KOY) in the AWE module for generating J–V curves.

### Integrating FO and AWE to make the FOWS_AWE_ system

The process design of the FOWS_AWE_ system is shown in Supplementary Fig. 17. Clean water was first extracted from the feed wastewater effluent tank via the FO process with the same setup as the independent FO module described earlier except the membrane area was 1 cm^2^. The extracted clean water diluted the KOH draw solution whose concentration was then recovered by splitting the water in the AWE using the diluted KOH as the electrolyte. The AWE outflow consisted of two KOH streams, one mixed with H_2_ and the other mixed with O_2_. The H_2_ and O_2_ were then separated from the KOH streams using a gravity-based gas–liquid separator, and the KOH stream free of gases flowed back to the draw solution tank. The KOH concentration was continuously monitored by analysing the change in the conductivity of the draw solution and the change in the pH of feed the wastewater effluent. Δ*C* was determined by subtracting the molar concentration of all impurities in the feed tank (Σ[impurities]_feed_) as listed in Supplementary Table 4 and the molar concentration of KOH in the feed tank due to RSF ([KOH]_feed_) from the molar concentration of recovered KOH in the draw solution tank ([KOH]_recovered_) as shown in Eq. 6:

6 $$\Delta C\,=\,{\left[{{{{{\rm{KOH}}}}}}\right]}_{{{{{{\rm{recovered}}}}}}}\,-\,\Sigma {\left[{{{{{\rm{impurities}}}}}}\right]}_{{{{{{\rm{feed}}}}}}}\,-\,{\left[{{{{{\rm{KOH}}}}}}\right]}_{{{{{{\rm{feed}}}}}}}$$

The separated H_2_ was collected by draining the water downwards in an inverted measuring cylinder, and the H_2_ production rate was determined by the water drainage volume over pre-determined time intervals. The H_2_ flux was then calculated by dividing the obtained H_2_ production rate by the FO membrane areas. The purity of the collected H_2_ was analysed by gas chromatography (990 Micro GC System, Agilent) equipped with a thermal conductivity detector. The obtained H_2_ production rates and H_2_ purity were used to determine the Faradaic efficiency (FE) of the system by dividing the experimentally measured H_2_ production rate (${r}_{{{{{\rm{{H}}}}}_{2}}}$) by the theoretical H_2_ production rate (${r}_{{{{{\rm{{H}}}}}_{2}},{{{{{\rm{ideal}}}}}}}$) (Eq. 7).

7 $${{{{{{\rm{FE}}}}}}}_{{{{{\rm{{H}}}}}_{2}}}\,=\,\frac{{r}_{{{{{\rm{{H}}}}}_{2}}}}{{r}_{{{{{\rm{{H}}}}}_{2}},{{{{{\rm{ideal}}}}}}}}\,=\,\frac{{r}_{{{{{\rm{{H}}}}}_{2}}}}{\frac{i}{2F}\,\times\,\frac{{RT}}{{P}_{0}}}$$

where *P*_0_ is atmospheric pressure (101 kPa), and *T* is the operating temperature (296 K). The SEC of the FOWS_AWE_ system (kWh Nm^–3^) was calculated using Eq. 8.

8 $${{{{{\rm{SEC}}}}}}\,=\,\frac{{i}_{{{{{{\rm{cell}}}}}}}\,\times\,U}{{r}_{{{{{\rm{{H}}}}}_{2}}}}$$

where *U* is the resulting voltage (V) obtained. The *i*_cell_ value was 1.55 A when deionised water was used as the feed source, 0.95 A when HK-WWE-1 was used as the feed source, and 0.90 A when HK-WWE-2 was used as the feed source during the long-term test.

### Supplementary information

Supplementary Information

Peer Review File

### Source data

Source data

## Data Availability

The data that supports the findings of the study are included in the main text and supplementary information files. Source data are provided with this paper.

## References

[1] IRENA. *Global Energy Transformation: A Roadmap to 2050* (International Renewable Energy Agency, 2019); Abu Dhabi; https://www.irena.org/publications/2019/Apr/Global-energy-transformation-A-roadmap-to-2050-2019Edition.

[2] Chi J, Yu H (2018). Water electrolysis based on renewable energy for hydrogen production. Chin. J. Catal..

[3] Chen Q, Kuang Z, Liu X, Zhang T (2022). Energy storage to solve the diurnal, weekly, and seasonal mismatch and achieve zero-carbon electricity consumption in buildings. Appl. Energy.

[4] IRENA. *Innovation Landscape Brief: Renewable Power-to-Hydrogen* (International Renewable Energy Agency, 2019); Abu Dhabi; https://www.irena.org/Publications/2019/Sep/Enabling-Technologies.

[5] IRENA. *Geopolitics of the Energy Transformation: The Hydrogen Factor* (International Renewable Energy Agency, 2022); Abu Dhabi; https://www.irena.org/publications/2022/Jan/Geopolitics-of-the-Energy-Transformation-Hydrogen.

[6] IRENA. *Global Hydrogen Trade to Meet the 1.5 °C ClimateGoal: Part III—Green Hydrogen Cost and Potential* (International Renewable energy Agency, 2022); Abu Dhabi; https://www.irena.org/Publications/2022/May/Global-hydrogen-trade-Cost.

[7] IEA. *Net Zero by 2050* (International Renewable Energy Agency, 2021); Paris; https://www.iea.org/reports/net-zero-by-2050.

[8] Odenweller A, Ueckerdt F, Nemet GF, Jensterle M, Luderer G (2022). Probabilistic feasibility space of scaling up green hydrogen supply. Nat. Energy.

[9] Hofste, R. et al. *Aqueduct 3.0: Updated Decision-Relevant Global Water Risk Indicators* (World Resources Institute, 2019); Washington DC; 10.46830/writn.18.00146.

[10] He C (2021). Future global urban water scarcity and potential solutions. Nat. Commun..

[11] Parkinson S (2021). Guiding urban water management towards 1.5 °C. npj Clean. Water.

[12] Yu L (2019). Non-noble metal-nitride based electrocatalysts for high-performance alkaline seawater electrolysis. Nat. Commun..

[13] Kuang Y (2019). Solar-driven, highly sustained splitting of seawater into hydrogen and oxygen fuels. Proc. Natl. Acad. Sci..

[14] Dresp S (2020). Efficient direct seawater electrolysers using selective alkaline NiFe-LDH as OER catalyst in asymmetric electrolyte feeds. Energy Environ. Sci..

[15] Lee B, Wang L, Wang Z, Cooper NJ, Elimelech M (2023). Directing the research agenda on water and energy technologies with process and economic analysis. Energy Environ. Sci..

[16] Kim J, Park K, Yang DR, Hong S (2019). A comprehensive review of energy consumption of seawater reverse osmosis desalination plants. Appl. Energy.

[17] McGovern RK, Lienhard V JH (2014). On the potential of forward osmosis to energetically outperform reverse osmosis desalination. J. Memb. Sci..

[18] Shaffer DL, Werber JR, Jaramillo H, Lin S, Elimelech M (2015). Forward osmosis: Where are we now?. Desalination.

[19] Cath T, Childress A, Elimelech M (2006). Forward osmosis: principles, applications, and recent developments. J. Memb. Sci..

[20] Yavuz, A. B., Karanikola, V., García-Payo, M. C. & Khayet, M. 9—Osmotic distillation and osmotic membrane distillation for the treatment of different feed solutions. In *Osmosis Engineering* 245–278 (Elsevier, 2021); 10.1016/B978-0-12-821016-1.00005-X.

[21] Veroneau SS, Nocera DG (2021). Continuous electrochemical water splitting from natural water sources via forward osmosis. Proc. Natl. Acad. Sci..

[22] Xie H (2022). A membrane-based seawater electrolyser for hydrogen generation. Nature.

[23] Rezaei M (2018). Wetting phenomena in membrane distillation: mechanisms, reversal, and prevention. Water Res..

[24] Lei Z (2020). Recent progress in electrocatalysts for acidic water oxidation. Adv. Energy Mater..

[25] Hou Y (2022). Strategies for electrochemically sustainable H_2_ production in acid. Adv. Sci..

[26] Jones E, Qadir M, van Vliet MTH, Smakhtin V, Kang S (2019). The state of desalination and brine production: a global outlook. Sci. Total Environ..

[27] Ansari AJ (2016). Factors governing the pre-concentration of wastewater using forward osmosis for subsequent resource recovery. Sci. Total Environ..

[28] Alrehaili O, Perreault F, Sinha S, Westerhoff P (2020). Increasing net water recovery of reverse osmosis with membrane distillation using natural thermal differentials between brine and co-located water sources: impacts at large reclamation facilities. Water Res..

[29] Fei, H. et al. Direct seawater electrolysis: from catalyst design to device applications. *Adv. Mater*. **2,** e2309211 (2023). 10.1002/adma.202309211 37918125

[30] Ge Q, Ling M, Chung T-S (2013). Draw solutions for forward osmosis processes: developments, challenges, and prospects for the future. J. Memb. Sci..

[31] Becker H (2023). Impact of impurities on water electrolysis: a review. Sustain. Energy Fuels.

[32] Achilli A, Cath TY, Childress AE (2010). Selection of inorganic-based draw solutions for forward osmosis applications. J. Memb. Sci..

[33] da Silva JRP (2021). Applicability of osmotic bioreactor using potassium pyrophosphate as draw solution combined with reverse osmosis for removal of pharmaceuticals and production of high quality reused water. J. Environ. Chem. Eng..

[34] Gilliam R, Graydon J, Kirk D, Thorpe S (2007). A review of specific conductivities of potassium hydroxide solutions for various concentrations and temperatures. Int. J. Hydrog. Energy.

[35] Nguyen HT (2015). A new class of draw solutions for minimizing reverse salt flux to improve forward osmosis desalination. Sci. Total Environ..

[36] Lu S (2018). Effect of aqueous electrolytes on the electrochemical behaviors of ordered mesoporous carbon composites after KOH activation as supercapacitors electrodes. J. Electroanal. Chem..

[37] Coronell O, Mariñas BJ, Zhang X, Cahill DG (2008). Quantification of functional groups and modeling of their ionization behavior in the active layer of FT30 reverse osmosis membrane. Environ. Sci. Technol..

[38] Arena JT, Chwatko M, Robillard HA, McCutcheon JR (2015). pH sensitivity of ion exchange through a thin film composite membrane in forward osmosis. Environ. Sci. Technol. Lett..

[39] Dionigi F, Reier T, Pawolek Z, Gliech M, Strasser P (2016). Design criteria, operating conditions, and nickel-iron hydroxide catalyst materials for selective seawater electrolysis. ChemSusChem.

[40] Mccutcheon JR, Elimelech M (2007). Modeling water flux in forward osmosis: Implications for improved membrane design. AIChE J..

[41] Zeng K, Zhang D (2010). Recent progress in alkaline water electrolysis for hydrogen production and applications. Prog. Energy Combust. Sci..

[42] Melián-Martel N, Sadhwani JJ, Malamis S, Ochsenkühn-Petropoulou M (2012). Structural and chemical characterization of long-term reverse osmosis membrane fouling in a full scale desalination plant. Desalination.

[43] Lee S, Boo C, Elimelech M, Hong S (2010). Comparison of fouling behavior in forward osmosis (FO) and reverse osmosis (RO). J. Memb. Sci..

[44] Yao M (2020). A review of membrane wettability for the treatment of saline water deploying membrane distillation. Desalination.

[45] Chang H (2021). A critical review of membrane wettability in membrane distillation from the perspective of interfacial interactions. Environ. Sci. Technol..

[46] Cho K, Hoffmann MR (2017). Molecular hydrogen production from wastewater electrolysis cell with multi-junction BiOx/TiO2 anode and stainless steel cathode: current and energy efficiency. Appl. Catal. B Environ..

[47] IRENA. *Green Hydrogen Cost Reduction: Scaling up Electrolysers to Meet the 1.5**°**C Climate Goal* (International Renewable Energy Agency, 2020); Abu Dhabi; https://www.irena.org/publications/2020/Dec/Green-hydrogen-cost-reduction.

[48] You S-J (2012). Temperature as a factor affecting transmembrane water flux in forward osmosis: steady-state modeling and experimental validation. Chem. Eng. J..

[49] Valladares Linares R (2016). Life cycle cost of a hybrid forward osmosis–low pressure reverse osmosis system for seawater desalination and wastewater recovery. Water Res..

[50] Hao X (2019). Environmental impacts of resource recovery from wastewater treatment plants. Water Res..

[51] Donald R, Boulaire F, Love JG (2023). Contribution to net zero emissions of integrating hydrogen production in wastewater treatment plants. J. Environ. Manag..

[52] Woods P, Bustamante H, Aguey-Zinsou K-F (2022). The hydrogen economy—Where is the water?. Energy Nexus.

[53] Boyd CE, Torrans EL, Tucker CS (2018). Dissolved oxygen and aeration in ictalurid catfish aquaculture. J. World Aquac. Soc..

[54] Colt J, Watten B (1988). Applications of pure oxygen in fish culture. Aquac. Eng..

[55] WRI. *Aqueduct Water Risk Atlas* (World Resources Institute, accessed on [03.2023]); (Washington DC, 2019) https://www.wri.org/data/aqueduct-global-maps-30-data.

[56] IEA. *Hydrogen Projects Database* (International Energy Agency, accessed on [03.2023]); (Paris, 2022) https://www.iea.org/reports/hydrogen-projects-database.

