# Supplementary material for: Ultra-fast green hydrogen production from municipal wastewater by an integrated forward osmosis-alkaline water electrolysis system
Source: Nat Commun. 2024 Mar 23;15:2617. doi: 10.1038/s41467-024-46964-8 (PMC10960855; doi:10.1038/s41467-024-46964-8)
Supplement: Supplementary file 1 — Supplementary Information [file 41467_2024_46964_MOESM1_ESM.pdf]

Supplementary Information for

Ultra-fast green hydrogen production from municipal wastewater by an integrated forward osmosis-alkaline water electrolysis system

Gabriela Scheibel Cassol[1], Chii Shang[1,2], Alicia Kyoungjin An[3], Noman Khalid Khanzada[3,4], Francesco Ciucci[5,6], Alessandro Manzotti[5], Paul Westerhoff[7], Yinghao Song[*,1], Li Ling[*,8]

1. Department of Civil and Environmental Engineering, The Hong Kong University of Science and Technology, Clear Water Bay, Kowloon, Hong Kong SAR, China
2. Hong Kong Branch of Chinese National Engineering Research Center for Control & Treatment of Heavy Metal Pollution, the Hong Kong University of Science and Technology, Clear Water Bay, Kowloon, Hong Kong SAR, China
3. School of Energy and Environment, City University of Hong Kong, Tat Chee Avenue Kowloon, Hong Kong SAR, China
4. NYUAD Water Research Center, New York University Abu Dhabi, P.O. Box 129188, Abu Dhabi, United Arab Emirates
5. Department of Mechanical and Aerospace Engineering, The Hong Kong University of Science and Technology, Clear Water Bay, Kowloon, Hong Kong SAR, China
6. Chair of Electrode Design for Electrochemical Energy Systems, University of Bayreuth, 95448 Bayreuth, Germany
7. Nanosystems Engineering Research Center for Nanotechnology-Enabled Water Treatment, School of Sustainable Engineering and The Built Environment, Arizona State University, Tempe, AZ, 85287 United States of America
8. Advanced Interdisciplinary Institute of Environment and Ecology, Beijing Normal University, 519087 Zhuhai, China

* Corresponding author: phone: (852) 9794 9274, e-mail: ysongat@connect.ust.hk; or phone: (852) 6353 0630, e-mail: lling@bnu.edu.cn.

List of Contents

Supplementary Note 1. Simulation of specific energy consumption at different KOH temperatures 5

Supplementary Note 2. Supply of the oxygen produced from $FOWS_{AWE}$ integrated systems to wastewater treatment plants 7

Supplementary Fig. 1. Electrolyser performance with varied electrolytes. The current density-voltage (J-V) curves for the electrolyser using potassium hydroxide (KOH), sodium bicarbonate ($NaHCO_3$), and potassium diphosphate ($K_4P_2O_7$) electrolytes under the same conditions. (Conditions: [KOH] = [$NaHCO_3$] = [$K_4P_2O_7$] = 1 M). 8

Supplementary Fig. 2. Effect of draw solution on water flux. Comparison of water flux using NaCl and KOH as the draw solution and pure water as feed of the FO process (Conditions: [KOH] = [NaCl] = 1 M, flow rate = 190 ml min^{-1}). 9

Supplementary Fig. 3. Effect of KOH draw solution on FO membrane structure. SEM images of (a) the support layer of a pristine TFC-FO membrane, and (b) the support layer of a 5-cycle used TFC-FO membrane using 1 M KOH as the draw solution and simulated wastewater effluent as the feed solution. 10

Supplementary Fig. 4. Effect of KOH draw solution on FO membrane chemistry. Fourier transform infrared spectroscopy (FTIR) absorbance spectra at wavenumbers of (a) 4000–2500 cm^{-1} and (b) 1800–400 cm^{-1} for the selective layer of a pristine TFC-FO membrane, compared to the FTIR absorbance spectra at wavenumbers of (c) 4000–2500 cm^{-1} and (d) 1800–400 cm^{-1} for the selective layer of a 5-cycle used TFC-FO membrane using 1 M KOH as draw solution. 11

Supplementary Fig. 5. Influence of different wastewater effluent feed solutions. The rejection rates of the TFC-FO membrane for feeding different wastewater samples in presence of a wide range of impurities. (Conditions: [KOH] = 1 M, S = 1 cm^2, operation duration = 5 h, i_{cell} = 1.05 A and 1.03 A for GD-WWE and NC-WWE, respectively, the species and content of impurity are detailed in Supplementary Table 4). 12

Supplementary Fig. 6. Influence of initial feed solution pH. The effect of initial wastewater effluent pH on impurity rejection rates in the FO process. Measurements were carried out using GD-WWE with the initial pH adjusted to 6.5, 7.5, and 8.5. (Conditions: [KOH] = 1 M, S = 1 cm^2, i_{cell} = 1.05 A). 13

Supplementary Fig. 7. Steady-state validation for the FOWS$_{AWE}$ system. The molar ratio of the H_2 generation rate to the water permeate rate using deionised water and real wastewater effluent as feed. (Conditions: [KOH] = 1 M, membrane area = 1 cm^2). 14
Supplementary Fig. 8. FO membrane structure after long-term operation. SEM images showing **a–b.** the selective layer of a pristine TFC-FO membrane and the TFC-FO membrane after 168 h of operation, respectively; **c–d.** the support layer of a pristine TFC-FO membrane and the TFC-FO membrane after 168 h of operation, respectively. (Conditions: [KOH] = 1 M, S = 1 cm^2, i_{cell} = 0.90 A for using HK-WWE2). 15
Supplementary Fig. 9. Hydrogen purity during long-term FOWS$_{AWE}$ operation. H_2 purity measurements during 168 hours of continuous FOWS$_{AWE}$ system operation. (Conditions: [KOH] = 1 M, S = 1 cm^2, i_{cell} = 0.90 A for using HK-WWE2). 16
Supplementary Fig. 10. Influence of initial feed solution pH. The effect of initial wastewater effluent pH on the water flux. Measurements over time were carried out using GD-WWE with the initial pH adjusted to 6.5, 7.5, and 8.5. (Conditions: [KOH] = 1 M, S = 1 cm^2, i_{cell} = 1.05 A). 17
Supplementary Fig. 11. Process performance using different wastewater effluent samples. a. Water flux; **b.** voltage, **c.** ΔC between feed and draw solution, and **d.** H_2 flux of the integrated FOWS$_{AWE}$ system when using different wastewater sources. (Conditions: [KOH] = 1 M, S = 1 cm^2, operation duration = 5 h, i_{cell} = 1.08 A, 1.05 A, and 1.03 A for HK-WWE2, GD-WWE, and NC-WWE, respectively). 18
Supplementary Fig. 12. FO membrane structure analysis. SEM images showing the selective layers of **a.** the pristine TFC-FO membrane, and **b.** the TFC-FO membrane after 5-h operation using GD-WWE. 19
Supplementary Fig. 13. Membrane performance comparison in the FOWS$_{AWE}$ system. The comparison in J_i (i_{cell} S^{-1}), H_2 flux, and K_wI between a TFC-FO membrane and a PTFE membrane using KOH as the operational solution of the FOWS$_{AWE}$ integrated system. (Conditions: [KOH] = 1 M, membrane area = 1 cm^2). 20
Supplementary Fig. 14. Electrolyser performance with different temperatures. The J-V curve performances for the FOWS$_{AWE}$ integrated system under 10°C, 20°C, 30°C, and 40°C. 21
Supplementary Fig. 15. The independent FO module for testing KOH as the draw solution of the FO process. a. Experimental process design; and **b.** the detailed setup of the FO cell. 22
Supplementary Fig. 16. The independent AWE module. The module consists in a 5-cell alkaline stack, an electrolyte supply tank, and a gas separator for collecting H_2 and O_2 for testing

electrolysis performance. **a.** Experimental process design, **b.** the setup of the 5-cell alkaline stack and its specification, and **c.** detailed design of the gas separator... 24

Supplementary Figure 17. Integrating the modules of FO and AWE. a. Experimental process design, and **b.** experimental setups of the $FOWS_{AWE}$ integrated systems. 25

Supplementary Table 1. The *Ji* and the voltage obtained from the J-V curve to simulate SEC at different temperatures. 26

Supplementary Table 2. Comparison of energy consumption for producing 1 m^3 of water... 27

Supplementary Table 3. EDX showing the C and O element and their ratio for the selective layer of a pristine TFC-FO membrane and the TFC-FO membrane after 168 h of operation... 28

Supplementary Table 4. Quality of the collected municipal wastewater effluents samples......29

Supplementary Note 1. Simulation of specific energy consumption at different KOH temperatures

The specific energy consumption (SEC) was calculated at temperatures of at 10°C, 20°C, 30°C, and 40°C. The first step in the SEC calculation was to determine the specific normalised current (J_i) at each temperature. J_i is defined as the current needed to equalise the water permeation rate through the membrane matches with the consumption rate of water being released in the form of hydrogen and oxygen gas in the electrolyser. J_i was calculated using Supplementary Equation 1 for each of the four temperatures.

$$J_i = \frac{i_{cell}}{S} = \left(\frac{K_w I}{d} RT \frac{2F}{FE\ N_{cell} V_m}\right) \Delta C = \Phi \cdot \Delta C \quad (1)$$

where Φ is the H_2 production potential (L A mol^{-1} cm^{-2}), K_w is membrane permeability coefficient (L atm^{-1} s^{-1} cm^{-1}), I is the van't Hoff factor, d is the membrane thickness, R is the ideal gas constant, T is the temperature, F is the Faraday's constant, N_{cell} is the number of cells constituting the electrolyser stack, and V_m is the molar volume of H_2O. The new J_i values were then used to determine the i_{cell} at each temperature by multiplying the J_i with the membrane surface area utilised (1 cm^2). The theoretical H_2 production rate (r_{H_2}) was then determined using the calculated i_{cell} values in Equation 2.

$$Q_{AWE} = i_{cell} \frac{FE \cdot N_{cell} V_m}{2F} \quad (2)$$

The next step was to determine the voltage value that corresponded to the calculated i_{cel} at each temperature. This was done by consulting the experimental current-voltage (J-V) curve, as shown in Supplementary Table 1. The identified voltage values were then input into Equation 3, along with the other required parameters to calculate the SEC for each temperature.

$$SEC = \frac{i_{cell} \times U}{r_{H_2}} \quad (3)$$

where U is the voltage (V) obtained from J-V curves in the previous sections.

Supplementary Note 2. Supply of the oxygen produced from $FOWS_{AWE}$ integrated systems to wastewater treatment plants

Supplying pure oxygen can substantially improve the efficiency of aerobic biological processes in wastewater treatment by raising dissolved oxygen levels and oxygen transfer rates, leading to higher treatment performance and reduced hydraulic residence times[1–3]. The oxygen demand for the aerobic process in a wastewater treatment plant can be estimated by multiplying the average influent wastewater flow rate by the average chemical oxygen demand (COD) of the wastewater, as showed in Equation 4.

$$O_2\ demand = \ Q\ \left(\frac{m^3}{day}\right) \times COD\left(\frac{kg}{m^3}\right) \quad (4)$$

where Q is the wastewater influent flow rate. As an example, a wastewater treatment plant receiving 50,000 m^3 day^{-1} of wastewater with a chemical oxygen demand of 400 mg L^{-1} would theoretically require approximately 20,000 kg of oxygen per day. Based on the stoichiometric ratio between oxygen and hydrogen (2:1 in molar basis) during electrolysis, we can calculate the equivalent mass of hydrogen to produce that amount of oxygen. For the example requiring 20,000 kg day^{-1} of oxygen, the equivalent mass of hydrogen would be 2,500 kg. This amount of hydrogen indicates the capacity needed for an electrolysis plant to meet the oxygen demand. Specifically, an electrolysis system with around 5-6 MW capacity would be capable of generating the required 2,500 kg of hydrogen per day to produce the needed 20,000 kg of oxygen daily.

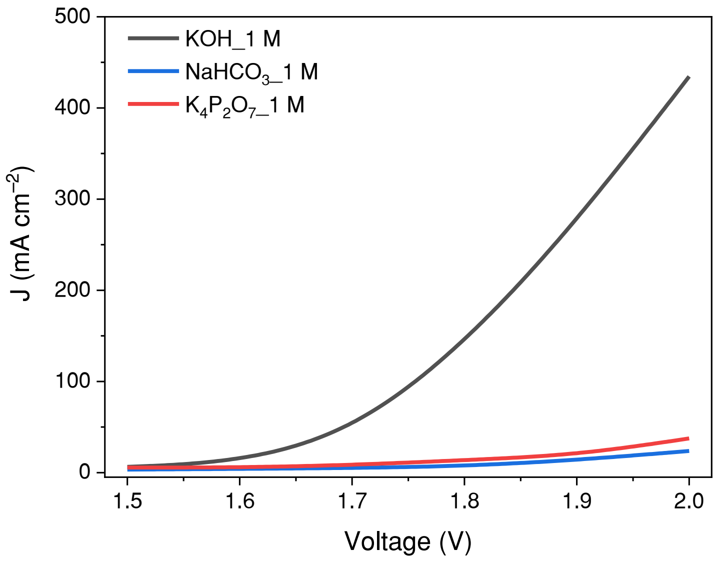

Supplementary Fig. 1. Electrolyser performance with varied electrolytes. The current density-voltage (J-V) curves for the electrolyser using potassium hydroxide (KOH), sodium bicarbonate ($NaHCO_3$), and potassium diphosphate ($K_4P_2O_7$) electrolytes under the same conditions. (Conditions: [KOH] = [$NaHCO_3$] = [$K_4P_2O_7$] = 1 M). Source data are provided as a Source Data file.

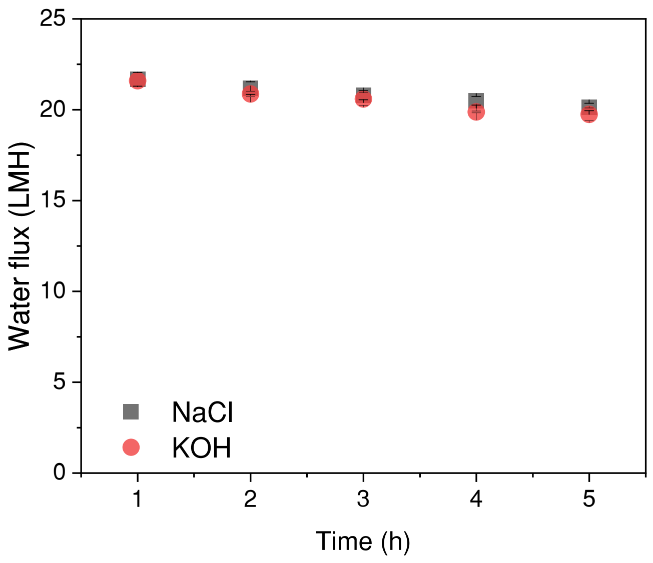

Supplementary Fig. 2. Effect of draw solution on water flux. Comparison of water flux using NaCl and KOH as the draw solution and pure water as feed of the FO process. Error bars represent standard deviation from three independent replicates. (Conditions: [KOH] = [NaCl] = 1 M, flow rate = 190 ml min^{-1}). Source data are provided as a Source Data file.

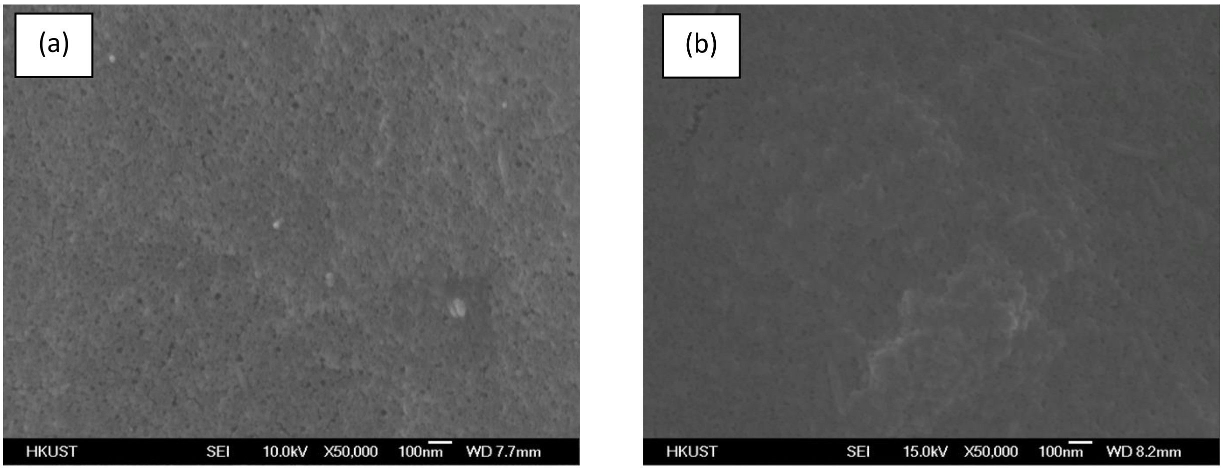

Supplementary Fig. 3. Effect of KOH draw solution on FO membrane structure. The scanning electron microscopy (SEM) images of (a) the support layer of a pristine TFC-FO membrane, and (b) the support layer of a 5-cycle used TFC-FO membrane using 1 M KOH as the draw solution and simulated wastewater effluent as the feed solution.

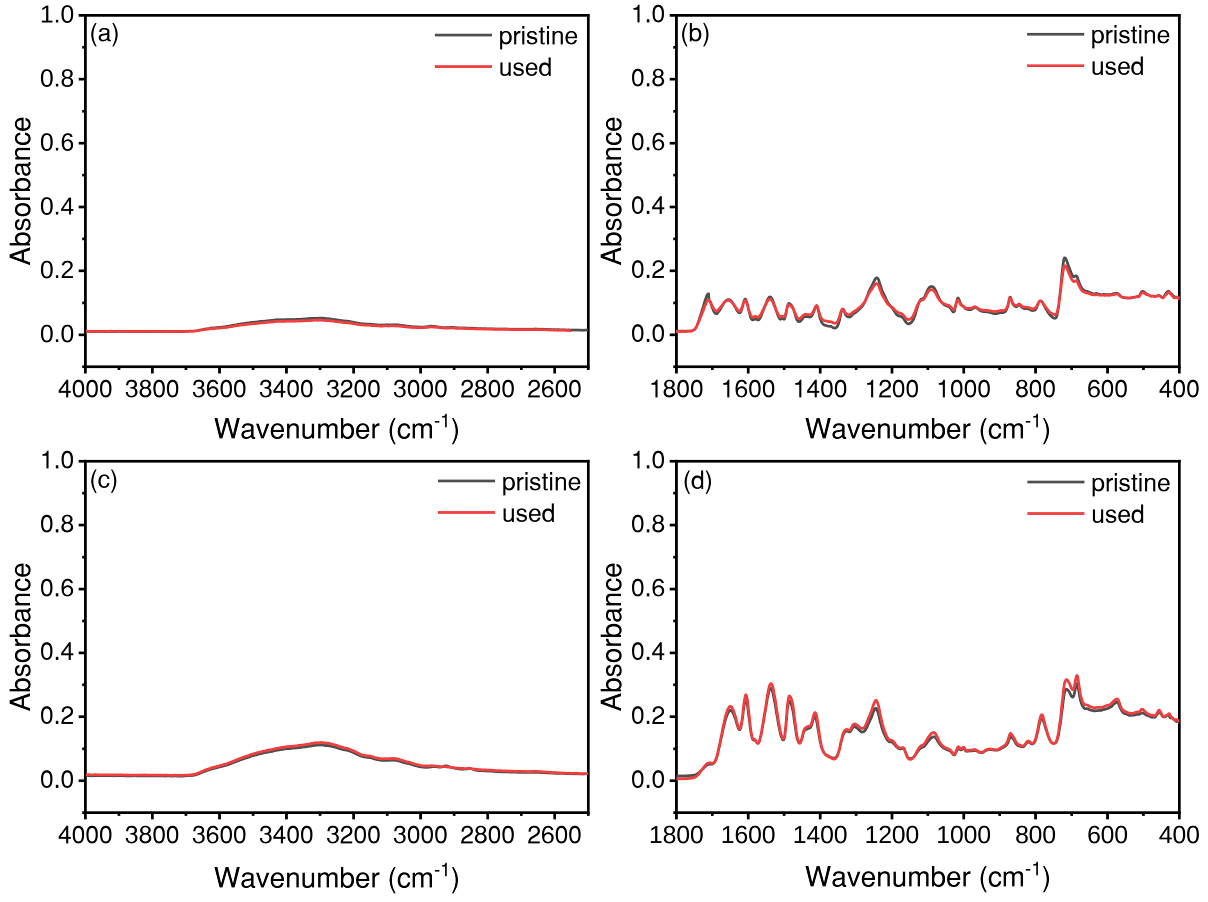

Supplementary Fig. 4. Effect of KOH draw solution on FO membrane chemistry. Fourier transform infrared spectroscopy (FTIR) absorbance spectra at wavenumbers of (a) 4000–2500 cm^{-1} and (b) 1800–400 cm^{-1} for the selective layer of a pristine TFC-FO membrane, and the FTIR absorbance spectra at wavenumbers of (c) 4000–2500 cm^{-1} and (d) 1800–400 cm^{-1} for the support layer of a 5-cycle used TFC-FO membrane using 1 M KOH as draw solution. Source data are provided as a Source Data file.

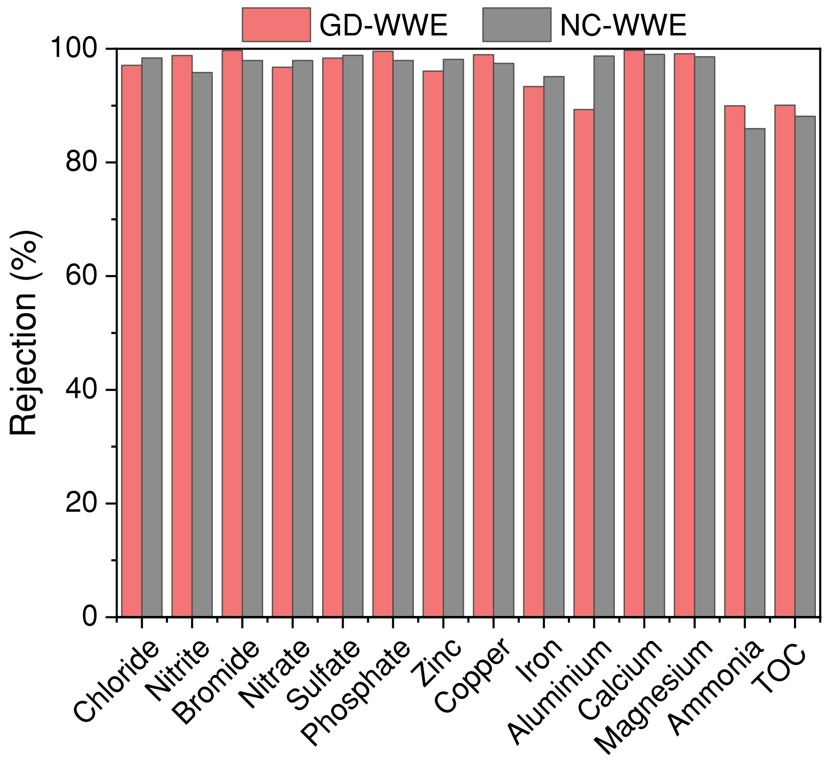

Supplementary Fig. 5. Influence of different wastewater effluent feed solutions. The rejection rates of the TFC-FO membrane for feeding different wastewater samples in presence of a wide range of impurities. (Conditions: [KOH] = 1 M, S = 1 cm^2, operation duration = 5 h, i_{cell} = 1.05 A and 1.03 A for GD-WWE and NC-WWE, respectively, the species and content of impurity are detailed in Supplementary Table 4). Source data are provided as a Source Data file.

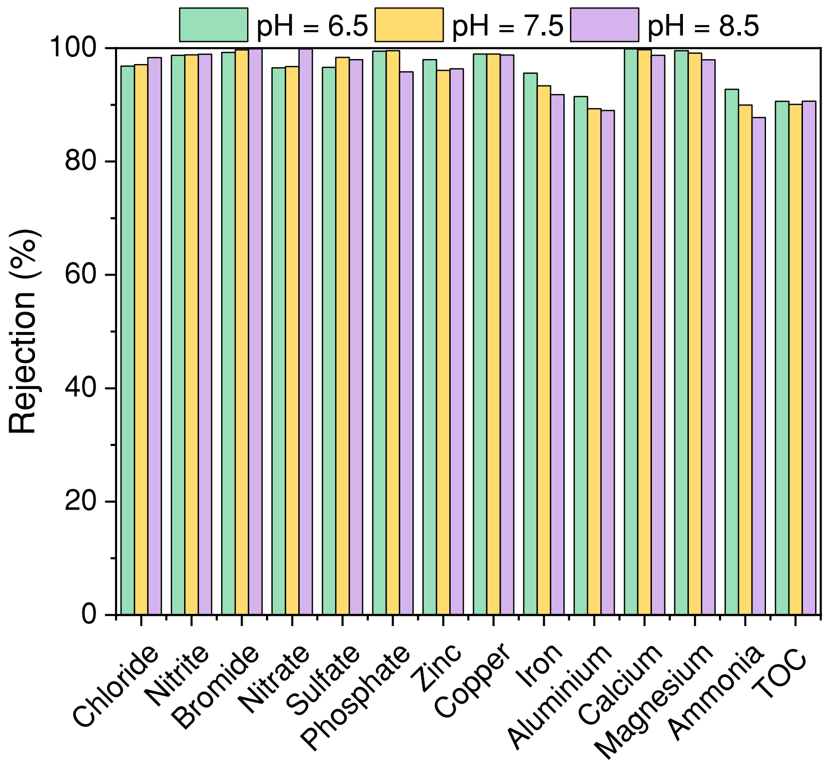

Supplementary Fig. 6. Influence of initial feed solution pH. The effect of initial wastewater effluent pH on impurity rejection rates in the FO process. Measurements were carried out using GD-WWE with the initial pH adjusted to 6.5, 7.5, and 8.5. (Conditions: [KOH] = 1 M, S = 1 cm^2, i_{cell} = 1.05 A). Source data are provided as a Source Data file.

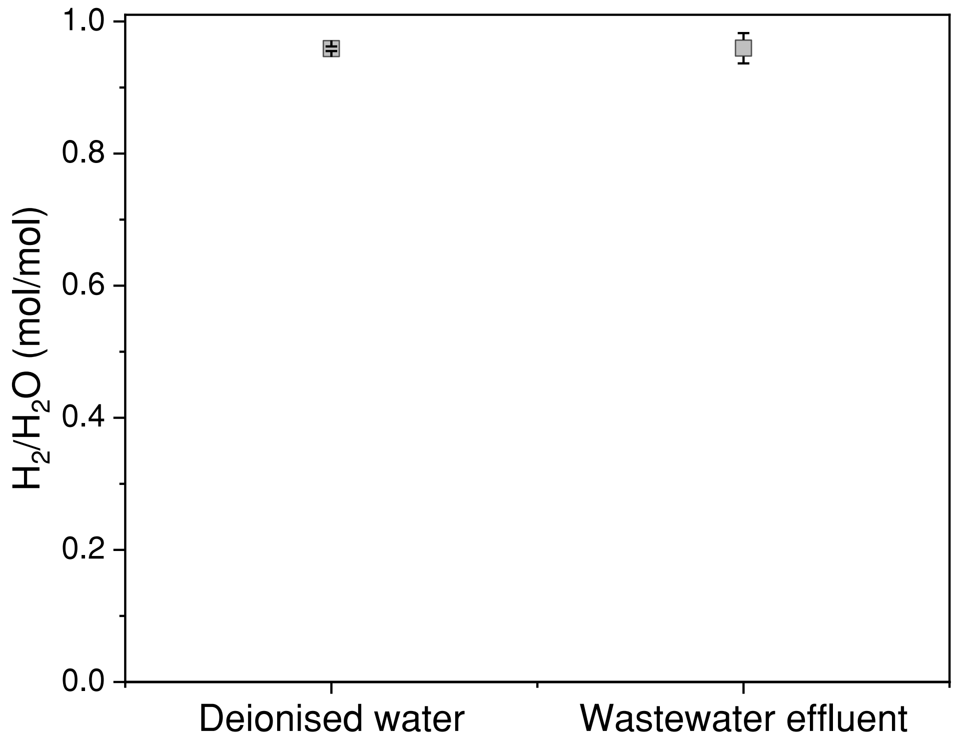

Supplementary Fig. 7. Steady-state validation for the FOWS$_{AWE}$ system. The molar ratio of the H_2 generation rate to the water permeate rate (H_2/H_2O) using deionised water and real wastewater effluent as feed. Error bars represent standard deviation from three independent replicates. (Conditions: [KOH] = 1 M, membrane area = 1 cm^2). Source data are provided as a Source Data file.

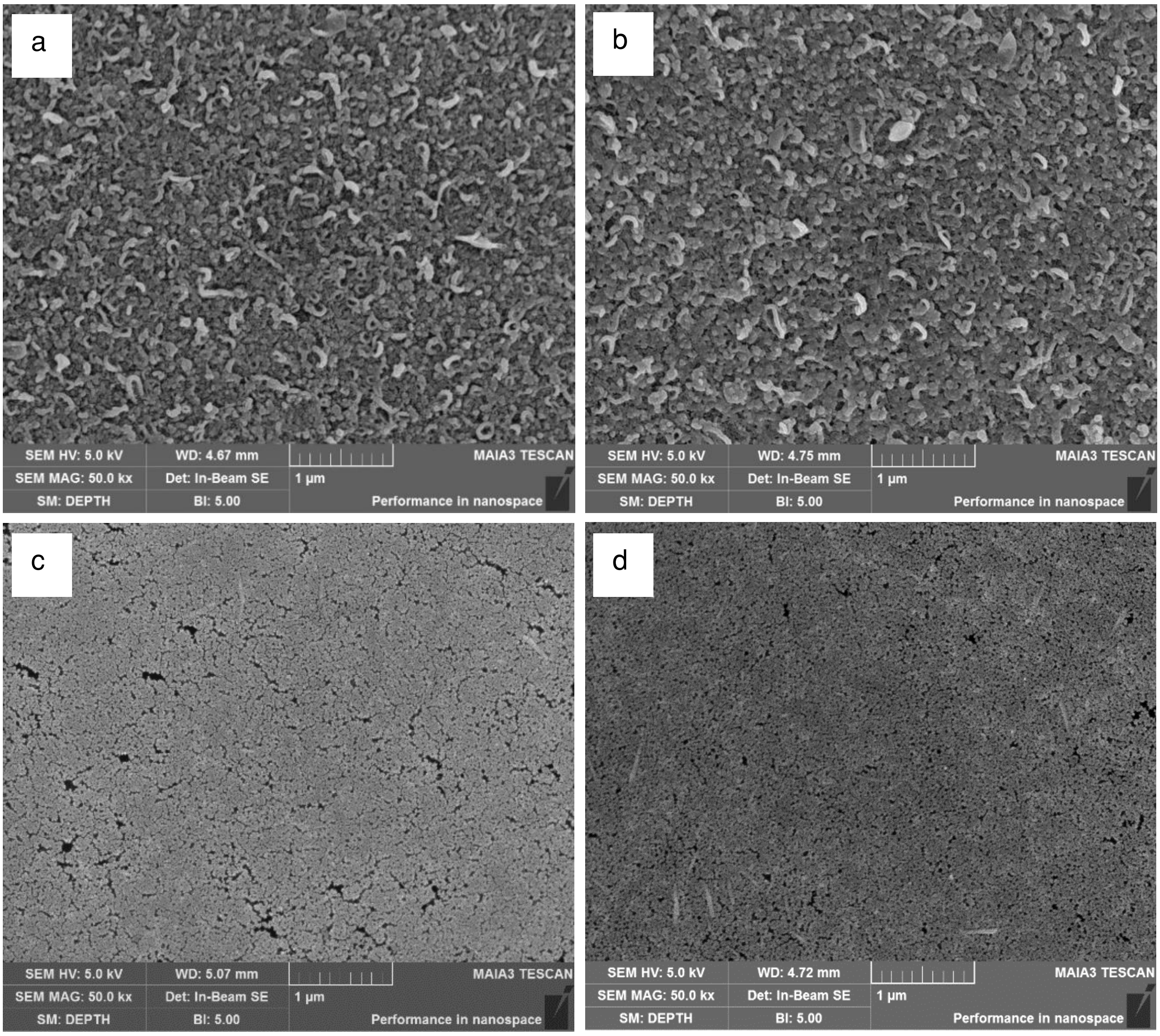

Supplementary Fig. 8. FO membrane structure after long-term operation. The SEM images showing **a–b.** the selective layer of a pristine TFC-FO membrane and the TFC-FO membrane after 168 h of operation, respectively; **c–d.** the support layer of a pristine TFC-FO membrane and the TFC-FO membrane after 168 h of operation, respectively. (Conditions: [KOH] = 1 M, S = 1 cm^2, i_{cell} = 0.90 A for using HK-WWE2).

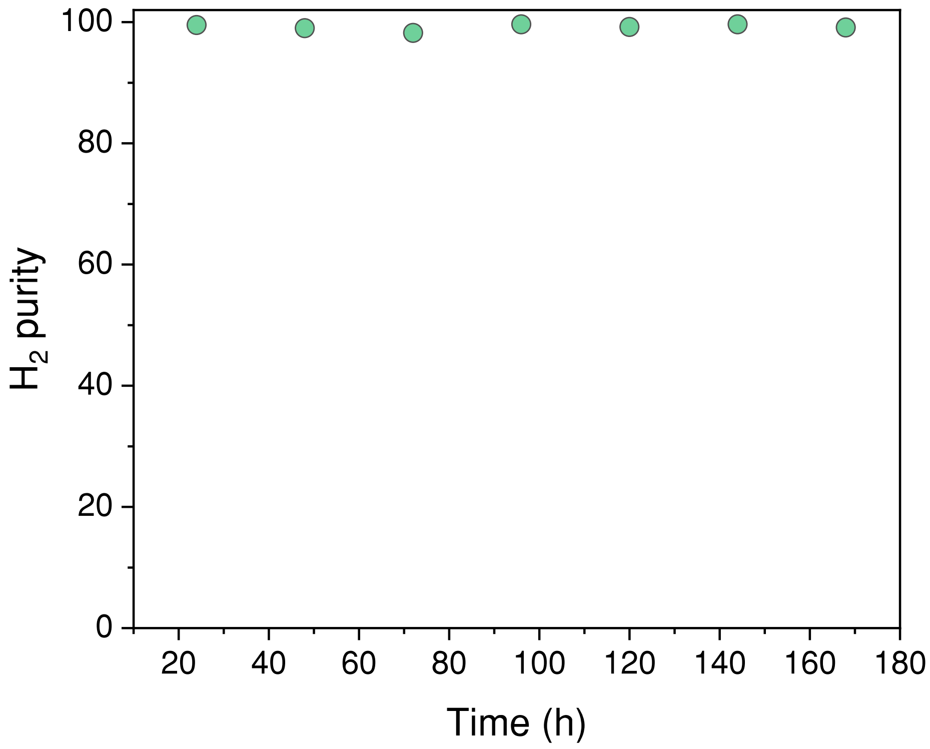

Supplementary Fig. 9. Hydrogen purity during long-term $FOWS_{AWE}$ operation. H_2 purity measurements during 168 hours of continuous $FOWS_{AWE}$ system operation. (Conditions: [KOH] = 1 M, S = 1 cm^2, i_{cell} = 0.90 A for using HK-WWE2). Source data are provided as a Source Data file.

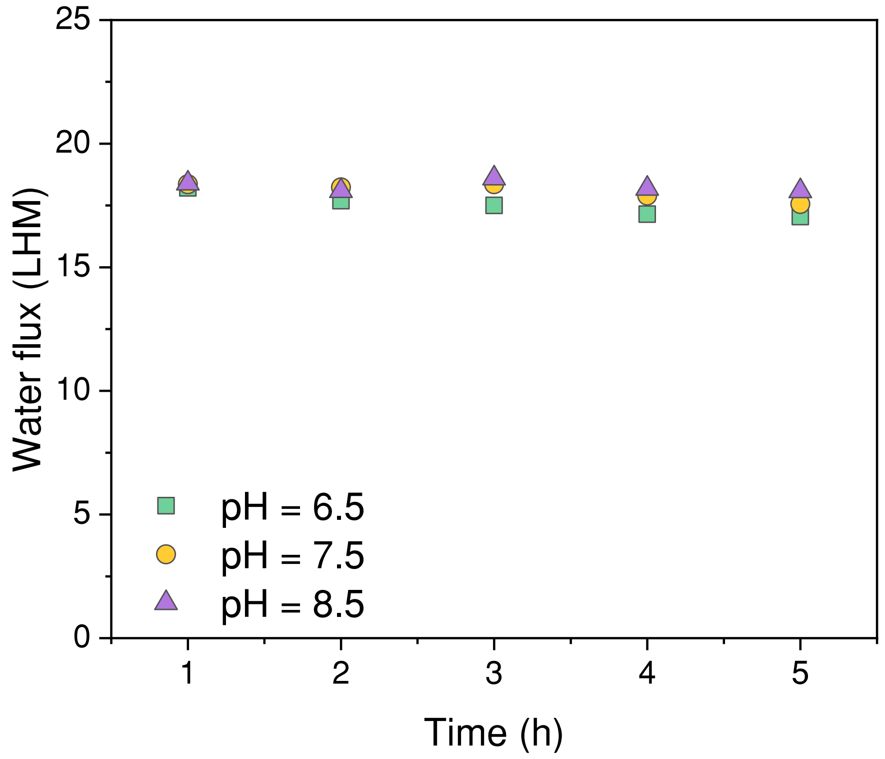

Supplementary Fig. 10. Influence of initial feed solution pH. The effect of initial wastewater effluent pH on the water flux. Measurements over time were carried out using GD-WWE with the initial pH adjusted to 6.5, 7.5, and 8.5. (Conditions: [KOH] = 1 M, S = 1 cm^2, i_{cell} = 1.05 A). Source data are provided as a Source Data file.

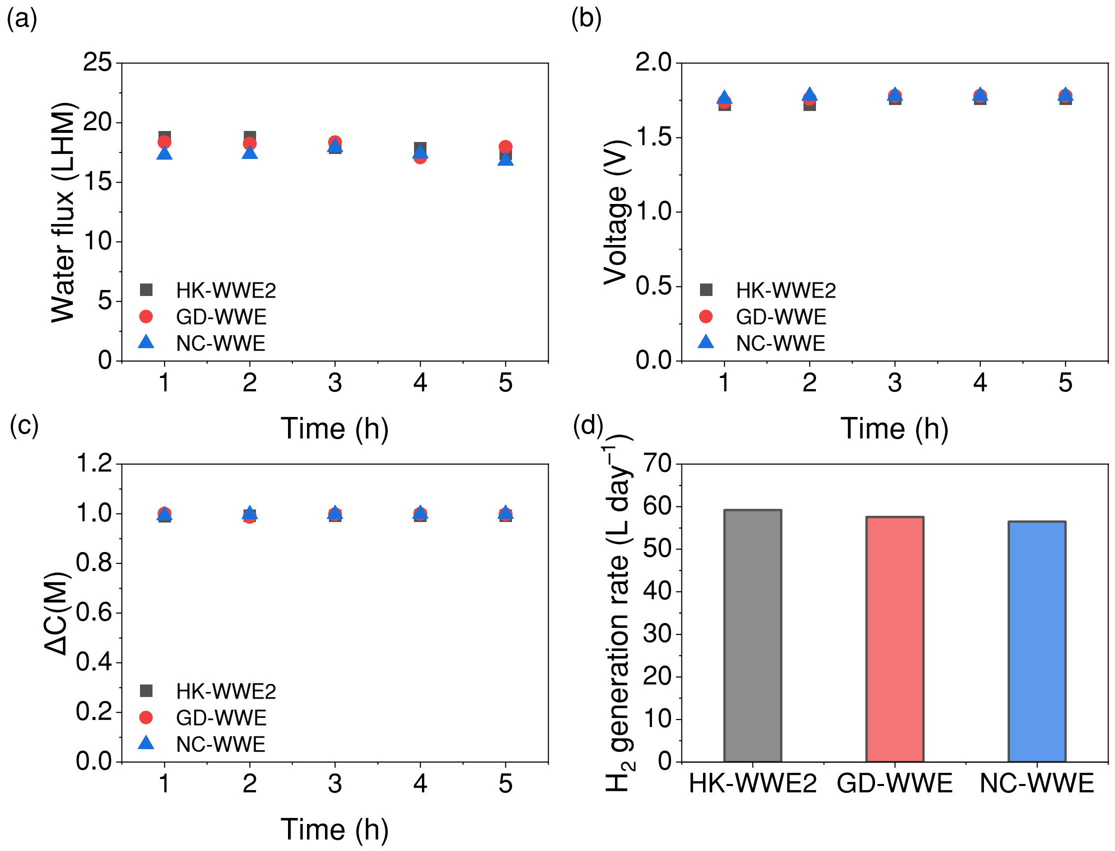

Supplementary Fig. 11. Process performance using different wastewater effluent samples. **a.** Water flux; **b.** voltage, **c.** concentration gradient (ΔC) between feed and draw solution, and **d.** H_2 flux of the integrated $FOWS_{AWE}$ system when using different wastewater sources. (Conditions: [KOH] = 1 M, S = 1 cm^2, operation duration = 5 h, i_{cell} = 1.08 A, 1.05 A, and 1.03 A for HK-WWE2, GD-WWE, and NC-WWE, respectively). Source data are provided as a Source Data file.

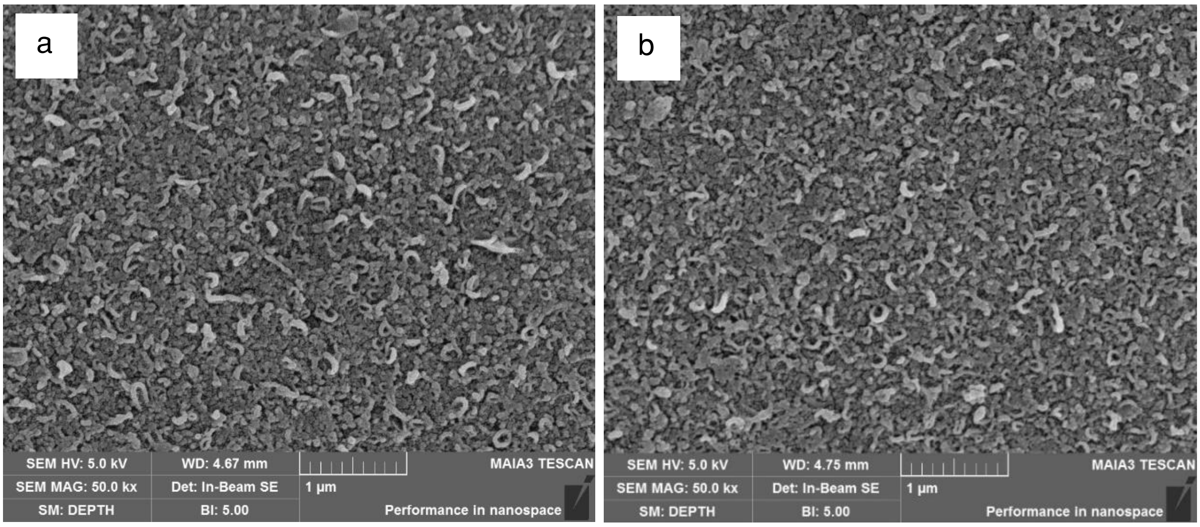

Supplementary Fig. 12. FO membrane structure analysis. SEM images showing the selective layers of **a.** the pristine TFC-FO membrane, and **b.** the TFC-FO membrane after 5-h operation using GD-WWE.

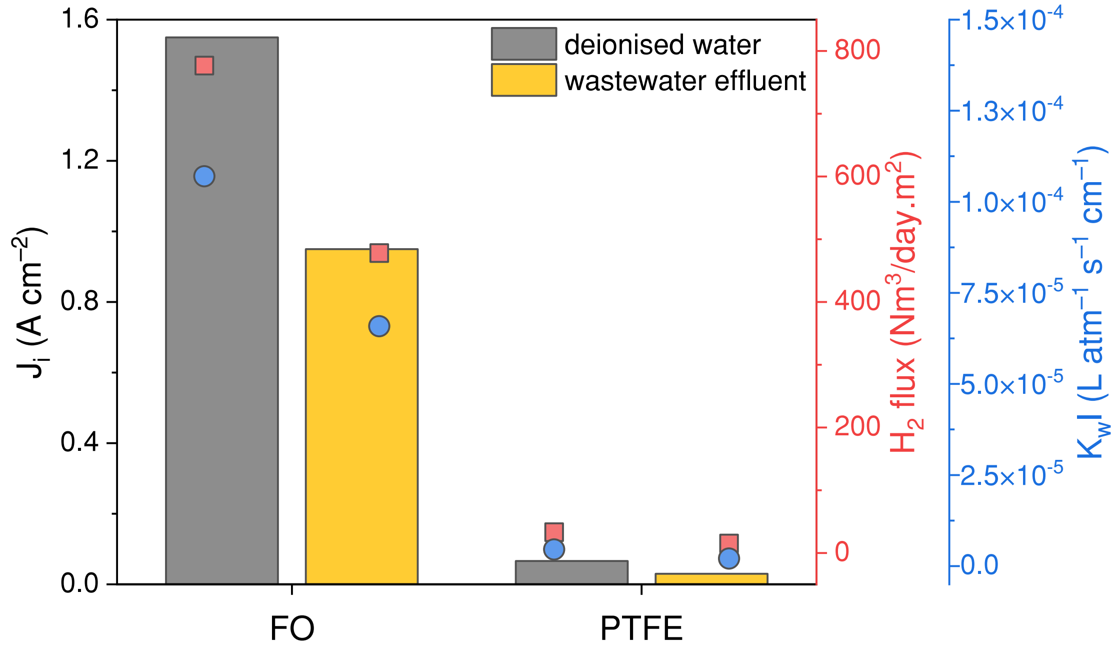

Supplementary Fig. 13. Membrane performance comparison in the $FOWS_{AWE}$ system. The comparison of the specific normalised current (J_i, i_{cell} S^{-1}), H_2 flux, and the product of the membrane permeability coefficient and van't Hoff factor (K_wI) between a TFC-FO membrane and a PTFE membrane using KOH as the operational solution of the $FOWS_{AWE}$ integrated system. (Conditions: [KOH] = 1 M, membrane area = 1 cm^2). Source data are provided as a Source Data file.

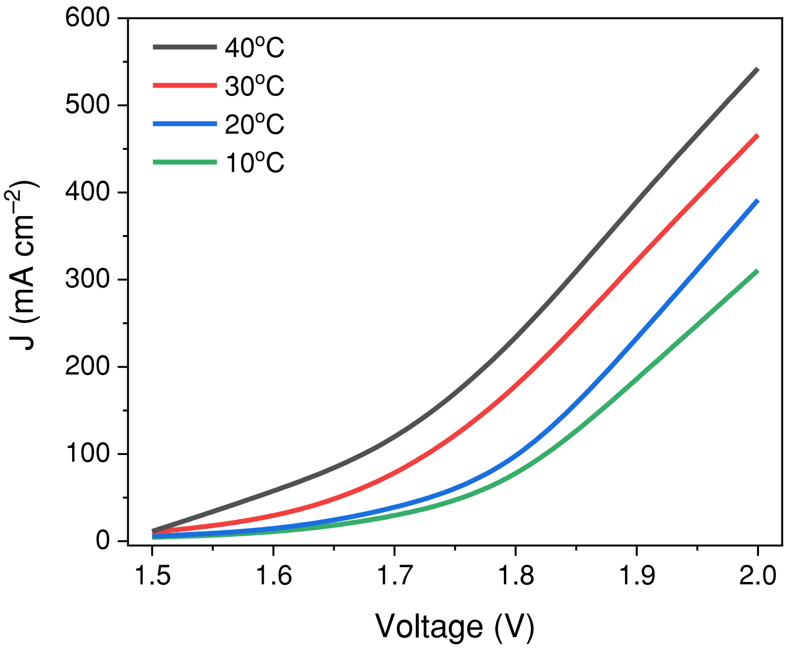

Supplementary Fig. 14. Electrolyser performance with different temperatures. The current density-voltage (J-V) curve performances for the FOWS$_{AWE}$ integrated system under 10°C, 20°C, 30°C, and 40°C. Source data are provided as a Source Data file.

(a)

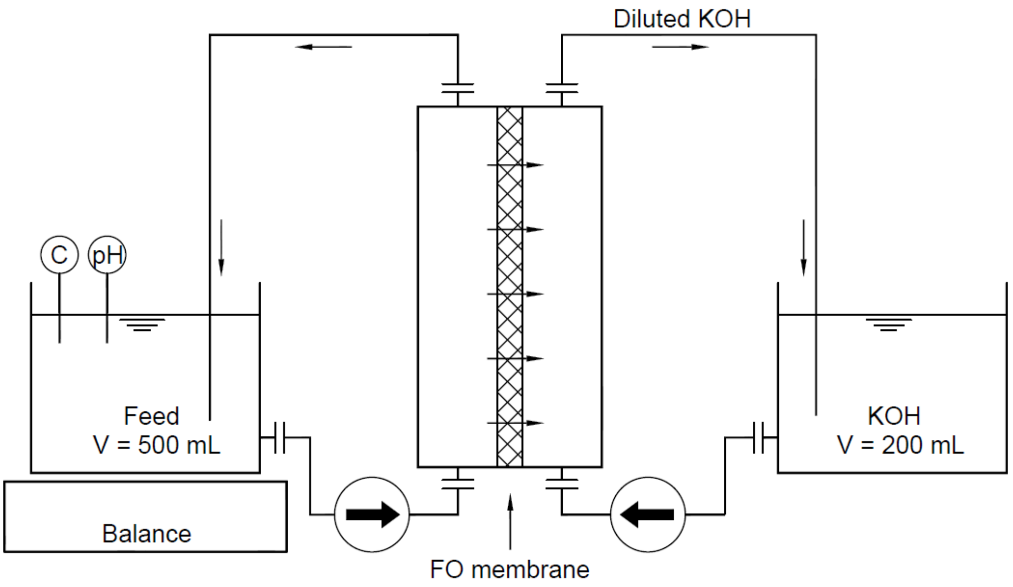

(b)

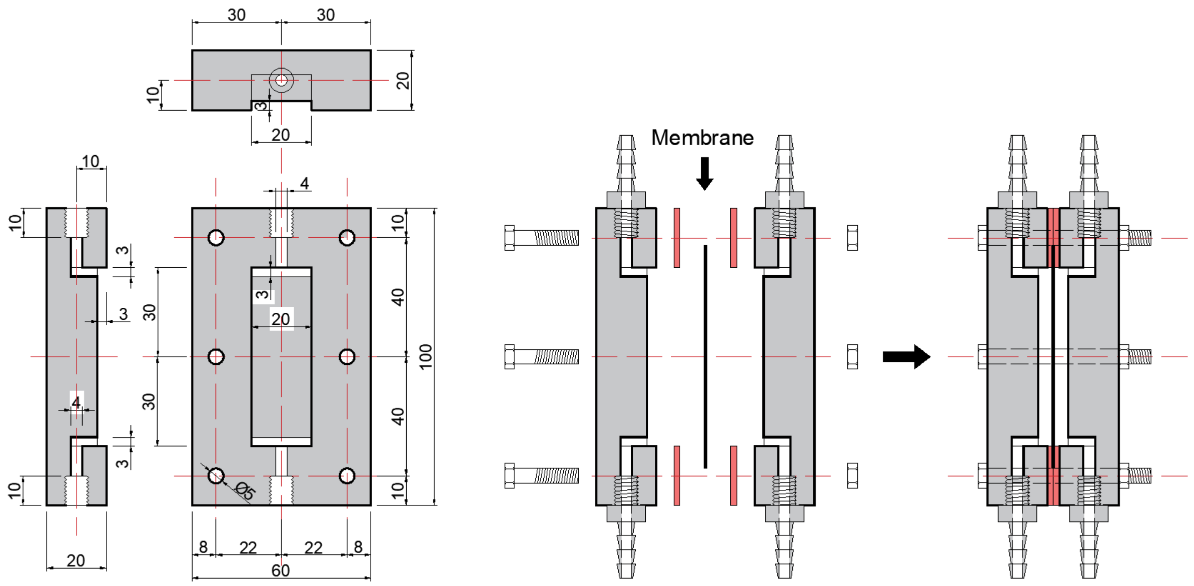

Supplementary Fig. 15. The independent FO module for testing KOH as the draw solution of the FO process. a. Experimental process design; and **b.** the detailed setup of the FO cell.

(a)

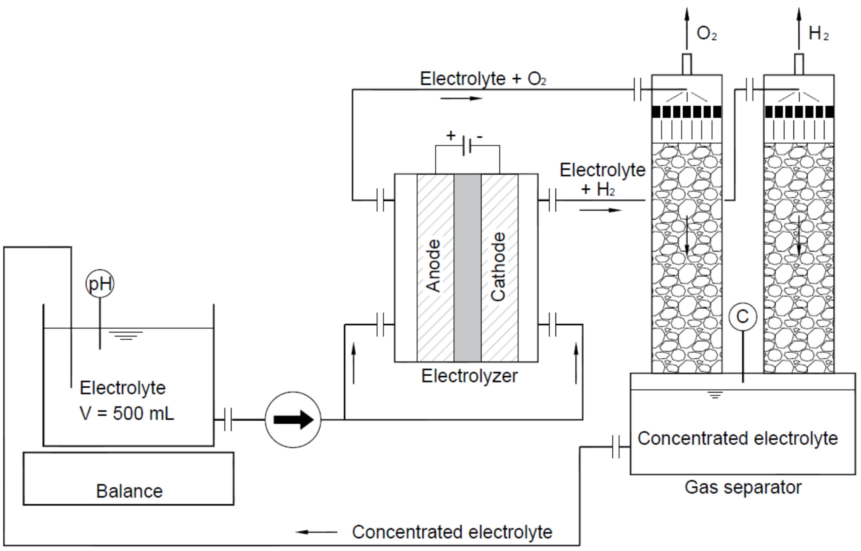
O2
H2
Electrolyte + O2
Electrolyte
+ H2
Anode
Cathode
Electrolyzer
pH
Electrolyte
V = 500 mL
Balance
C
Concentrated electrolyte
Gas separator
Concentrated electrolyte

(b)

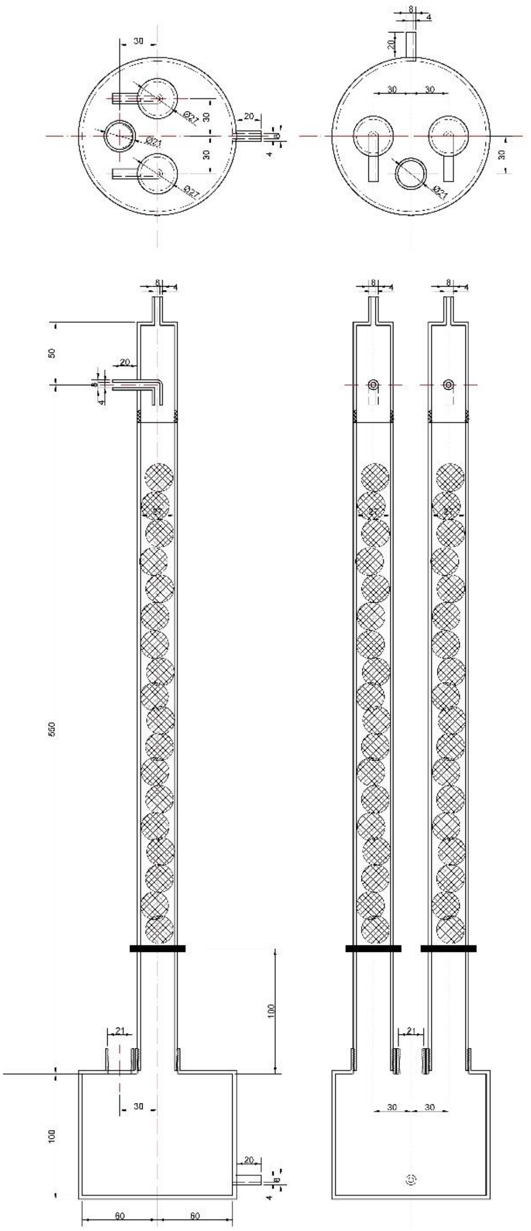

Supplementary Fig. 16. The independent AWE module. The module consists in a 5-cell alkaline stack, an electrolyte supply tank, and a gas separator for collecting H_2 and O_2 for testing electrolysis performance. **a.** Experimental process design, and **b.** the detailed design of the gas separator.

(a)

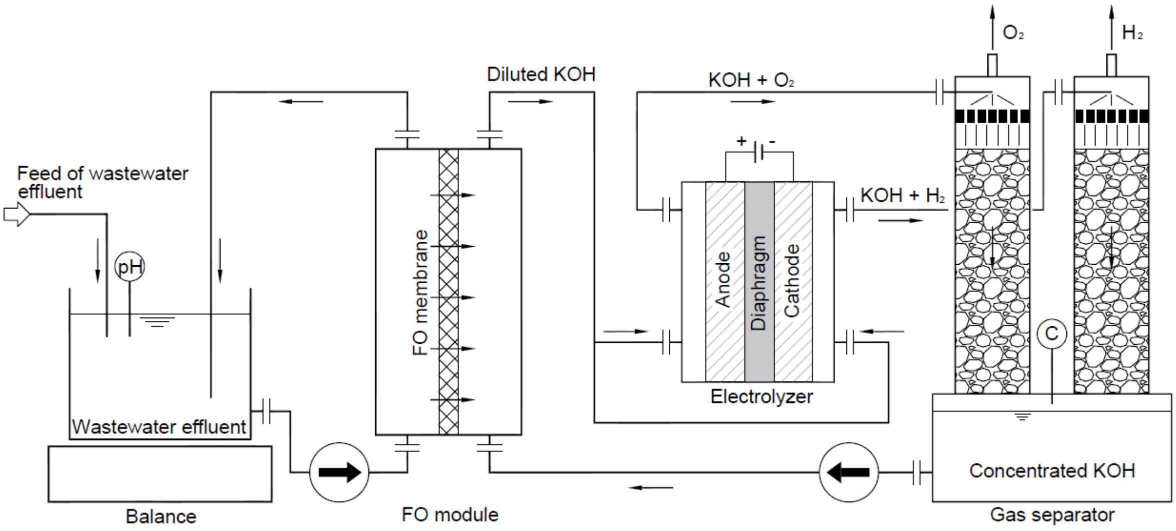

(b)

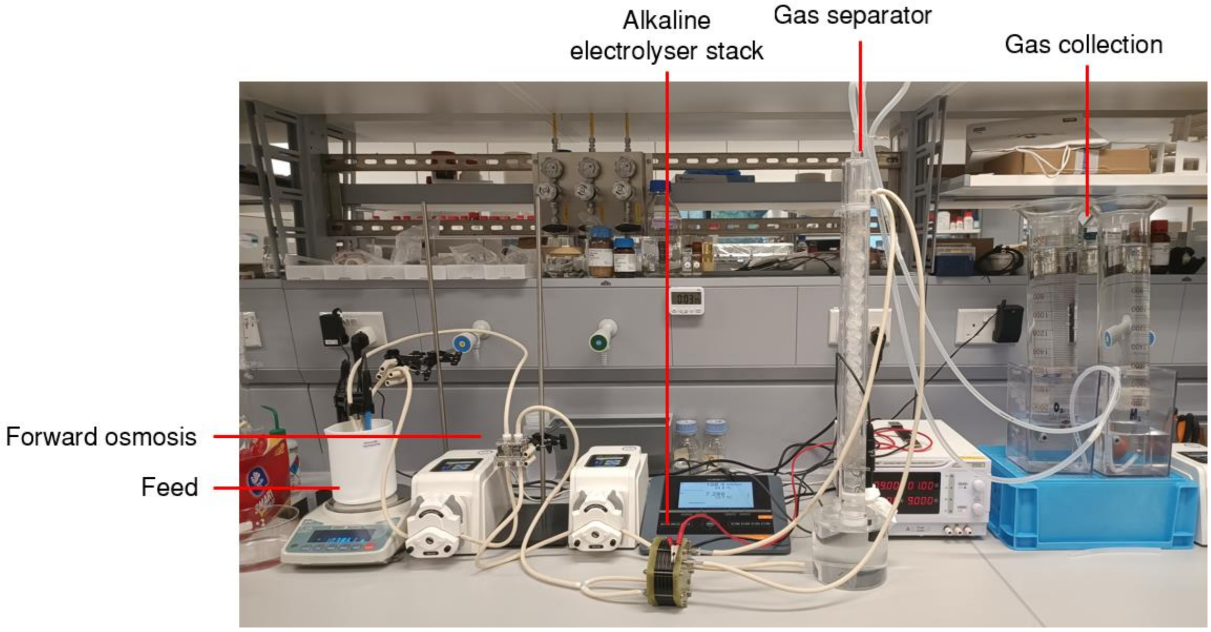

Supplementary Figure 17. Integrating the modules of FO and AWE. a. Experimental process design, and **b.** experimental setups of the $FOWS_{AWE}$ integrated systems.

Supplementary Table 1. The specific normalised current (*Ji*) and the resulting voltage obtained from the J-V curve to simulate SEC at different temperatures.

**Temperature**	**J_i (A m^{-2})**	**Voltage (V)**
10°C	0.91	1.85
20°C	0.94	1.80
30°C	0.98	1.72
40°C	1.01	1.65

Supplementary Table 2. Comparison of energy consumption for producing 1 m^3 of water.

Reference	Process	kWh m^{-3}
(McGovern and Lienhard V, 2014)	Two-pass reverse osmosis	3.48[1]
(McGovern and Lienhard V, 2014)	Forward osmosis with draw solute regeneration	3.58[2]
(McGovern and Lienhard V, 2014)	Forward osmosis without draw solute regeneration	0.10[3]

[1]Assuming 47% efficiency for the first RO pass[4].

[2]Assuming 47% efficiency for draw solute (NaCl) regeneration via RO[4].

[3]Estimation of pumping power consumption[4].

Supplementary Table 3. EDX showing the C and O element and their ratio for the selective layer of a pristine TFC-FO membrane and the TFC-FO membrane after 168 h of operation.

**TFC-FO membrane**	**Element**	**Mass Norm (%)**	**Atom (%)**	**Mass Norm (%)**
	C	82.08	85.92	3.80
Pristine	O	17.92	14.08	1.00
	C/O Ratio	4.58	6.10	/
	C	80.00	84.18	3.59
After168-h use	O	20.00	15.82	0.92
	C/O Ratio	4.00	5.32	/

Supplementary Table 4. Quality of the collected municipal wastewater effluents samples.

Parameter	HK-WWE1(i)	HK-WWE2(ii)	GD-WWE(iii)	NC-WWE(iv)
Conductivity (mS cm^{-1})	N.M.*	0.89	4.87	1.44
TOC (mg L^{-1} as C)	5.23	18.21	20.53	44.36
pH	7.2	7.85	7.42	7.29
Color (PtCo)	N.M.*	46	86	37
Ammonia (mg L^{-1} as N)	1.09	0.48	0.59	0.12
Chloride (mg L^{-1})	150.2	1.27	9.42	2.56
Nitrite (mg L^{-1})	0.37	0.03	0.01	0.14
Bromide (mg L^{-1})	N.D.*	N.D.*	0.03	0.02
Nitrate (mg L^{-1})	4.43	0.05	0.37	0.28
Sulfate (mg L^{-1})	N.M.*	0.23	1.40	0.68
Phosphate (mg L^{-1})	N.M.*	0.19	0.38	0.22
Calcium (mg L^{-1})	N.M.*	29.24	74.25	101.14
Magnesium (mg L^{-1})	N.M.*	4.35	110.78	28.87
Zinc (mg L^{-1})	N.M.*	0.07	0.06	N.D.
Copper (mg L^{-1})	N.M.*	0.16	0.16	0.06
Iron (mg L^{-1})	N.M.*	0.10	0.09	0.05
Aluminium (mg L^{-1})	N.M.*	0.18	0.15	0.07

*N.D.: not detected

*N.M.: not measured

*(i) HK-WWE1: wastewater effluent collected from Hong Kong; (ii) HK-WWE2: wastewater effluent collected from Hong Kong; (iii) GD-WWE: wastewater effluent collected from Guangdong province; and (iv) NC-WWE: wastewater effluent collected from Northeast China.

Supplementary references

1. Donald, R., Boulaire, F. & Love, J. G. Contribution to net zero emissions of integrating hydrogen production in wastewater treatment plants. *J. Environ. Manage.* **344**, 118485 (2023).
2. Woods, P., Bustamante, H. & Aguey-Zinsou, K.-F. The hydrogen economy - Where is the water? *Energy Nexus.* **7**, 100123 (2022).
3. Skouteris, G., Rodriguez-Garcia, G., Reinecke, S. F. & Hampel, U. The use of pure oxygen for aeration in aerobic wastewater treatment: A review of its potential and limitations. *Bioresour. Technol.* **312**, 123595 (2020).
4. McGovern, R. K. & Lienhard V, J. H. On the potential of forward osmosis to energetically outperform reverse osmosis desalination. *J. Memb. Sci.* **469**, 245–250 (2014).
